# Bayesian adaptive trial designs for evaluating low-risk programmatic changes for quality improvement in health services: a simulation study

**DOI:** 10.1186/s12874-026-02780-w

**Published:** 2026-02-27

**Authors:** Min Jung Kim, David Prieto-Merino, Jennifer Nicholas, Luke Allen, Andrew Bastawrous, David Macleod

**Affiliations:** 1 Faculty of Epidemiology and Population Health, London School of Hygiene & Tropical Medicine, London, UK; 2 Universidad de Alcalá, Madrid, Spain; 3 International Centre for Eye Health, Clinical Research Department, London School of Hygiene & Tropical Medicine, London, UK; 4 Present Address: Biomedical Research Institute, Seoul National University Hospital, Seoul, Republic of Korea

**Keywords:** Simulation, Bayesian analysis, Adaptive trial, Interim analysis, Stopping rules, Quality improvement

## Abstract

**Background:**

Quality improvement in health service programmes often involves introducing small, low-risk programmatic changes, such as modifying workflows, to incrementally improve outcomes, which accumulate over time to have significant overall gains in quality and efficiency. Although these changes are common in health services, they are rarely evaluated using statistically rigorous designs, partly because conventional randomised trials are perceived as inefficient for detecting modest effects. This study was motivated by a vision screening and referral programme and focuses on evaluating the modest but important impacts of low-risk QI interventions.

**Methods:**

To advance data-driven approaches for achieving quality improvement, we used a simulation study to explore the use of Bayesian adaptive trial designs to compare two variants of programmatic changes that yield small improvements in outcomes. The study examined key adaptive design features, including interim analysis frequency, prior specification, and early stopping rules for efficacy and equivalence. Changes in error rates, sample size, and bias were assessed across scenarios with small effect sizes ranging from 0% to 5%.

**Results:**

The findings were used to configure an ideal trial design that prioritises rapid identification of the more effective programmatic change while minimizing the risk of adopting an inferior one. The recommended trial design incorporates a sceptical prior, a stringent stopping rule for efficacy, and a relaxed criteria to stop for equivalence. Under this design, a marginal improvement as small as 1% could be detected with high probability using considerably fewer participants than would be required under conventional, fixed-size randomised controlled trials.

**Conclusions:**

Bayesian adaptive trial designs offer a feasible approach for evaluating low-risk, incremental QI interventions in high-throughput service settings. Their use may support more efficient, data-driven decision-making when modest improvements are expected and the consequences of incorrect adoption are limited.

**Supplementary Information:**

The online version contains supplementary material available at 10.1186/s12874-026-02780-w.

## Background

Health service programmes often fall short of their desired standards, preventing the delivery of high-quality care and equitable access. To address this, quality improvement (QI) is essential for continuously enhancing both efficiency and equity in health care [1, 2]. QI efforts can range from routine, experience-driven activities – such as sending reminder messages or adjusting clinic schedules to improve attendance – to more research-driven initiatives. The research aspect of QI has been of a particular interest in health services research, aiming to ensure that the QI efforts are ground in robust evidence [3, 4].

However, two major challenges arise in QI research. First, the impact of individual programmatic change is often modest and context-specific. QI typically involves incremental adjustments, each yielding small gains, with the expectation that these will cumulatively produce a large overall impact [5]. The use of randomised controlled trials (RCTs) can be challenging in this context because the effect sizes are uncertain and determining appropriate sample sizes are difficult. In fact, pragmatic RCTs are rarely performed to assess programmatic changes. Instead, non-experimental approaches – such as anecdotal evidence, case reports, or before-and-after studies – are common [6–8], but they are prone to confounding and limit external validity. This leads to the second challenge: there is often a disconnect between the quality of evidence needed for effective QI and the limitations of the research methods commonly used in these contexts. Inadequate quality and quantity of evidence acts as a limiting factor for QI [7].

In other areas of clinical research, adaptive trials have emerged as flexible and efficient study designs. By monitoring accumulating data through interim analyses, adaptive features – such as arm dropping and early stopping – improves responsiveness and streamlines the trial procedure [9]. These designs have been widely used in areas like Covid-19 vaccine development, oncology, and other pharmaceutical studies [10–14], demonstrating the potential to their scope beyond the current applications. Yet, directly transferring trial methodologies from clinical trials is not straightforward, as QI efforts often differ in priorities, resource availability, and other operational characteristics.

This underscores the need to understand how adaptive designs can be integrated into health service programmes. Unlike traditional clinical trials, QI interventions are usually low-cost and low-risk. Yet, randomized evaluation of QI interventions are usually avoided because conventional trial designs are perceived as too time-consuming. We propose adopting Bayesian adaptive trial designs as a feasible alternative to the non-randomised methods that currently dominate in QI research. Such trial designs can be configured to detect even small programmatic effects while maintaining acceptable operating characteristics to produce reliable results and inform decision-making. To explore this, we conducted a series of simulations to evaluate the impact of key design features, including early stopping thresholds and number and timing of interim analyses. Specifically, we aimed to assess how adaptive trials can be configured to balance key operating characteristics, such as error rates and sample size, within acceptable limits of QI research. The next section describes the setup of our simulation study, including the scenarios tested and the adaptive design features explored, followed by results and a discussion on their potential application in QI efforts.

## Methods

### Motivating example

This study was motivated by a QI initiative within a health service programme aimed at reducing vision impairment (VI) by promoting early identification and timely access to care. This vision screening programme uses a smartphone-based screening app and a cloud-based data system to identify individuals with VI and refer them to appropriate ophthalmology services. Since first launched in 2018, the programme has screened over 12.4 million people in 12 countries, identifying 2.4 million with VI. A cluster RCT in Kenya demonstrated its effectiveness, doubling referral uptake from 22% with standard care to 54% with the programme [15].

Yet, internal data shows that the only a half of those identified with VI attend their referral appointments, representing a critical gap in care continuity. To address this, the programme aimed to develop a QI framework for improving patient attendance at ophthalmology appointments. patient journey through care. The research team proposed potential solutions that involved simple, small-scale programmatic changes, such as adjusting the content or frequency of reminder messages or adding images to referral cards. But the optimal implementation – such as timing (morning vs. evening) or message wording, or which of the two potential images to put on a card – may be uncertain. In this context, there was a need to develop a structured approach to compare different variants of a programmatic change to identify the more effective variant, even if only marginally better, to adopt into the programme.

### Aims

This simulation study was modelled after a health service programme similar to the vision screening programme, where patients are followed to a binary outcome, such as clinic attendance or referral uptake. In this context, a *programmatic change* was defined as any low-cost, low-risk intervention for improving the probability of a successful outcome. It was envisioned that this QI effort would operate in high-throughput, routine data settings where outcomes are observed shortly after randomisation.

Our aim was to explore how Bayesian adaptive trial designs could be configured as a feasible alternative that maintains statistical rigour when comparing two programmatic variants with small or no differences in effect. Through simulations, we assessed trial performance across various scenarios with different effect sizes and reported the error rates, sample size, coverage, and bias of these trial designs. The results were used to recommend trial configurations that achieve a balance between statistical rigour and practical feasibility. The findings are intended to guide the design of adaptive trials for QI research for implementation in programmes such as the vision screening programme, while offering more robust evidence than non-randomised methods.

### Data-generating mechanisms

Our data modelled a comparison between two variants (arm A and arm B) across six scenarios, with absolute effect differences of 0%, 1%, 2%, 3%, 4%, and 5%. A 0% difference represented no effect, while a 5% difference represented a modest improvement by the superior variant. We did not consider larger effect differences to focus on detecting marginal improvements.

We fixed the probability of success in arm A at 50%. For arm B, we varied probabilities of success between 50% and 55%. We set the baseline probability of success around 50% to maximize the variability in the data and ensure that the sample sizes estimated would be sufficient to detect similar effect differences if the true baseline probabilities were different to 50%.

We generated 1,000 datasets for each of the six scenarios, which was deemed sufficient to assess the performance measures outlined below with Monte Carlo errors accounting for the randomness of the simulations [16]. For example, 1,000 simulations were deemed adequate to estimate a 90% likelihood of correctly identifying a superior arm, with a Monte Carlo error of 0.95%. It also enabled an adequate estimation of 95% coverage with a Monte Carlo error of 0.7%.

### Model structure

Each simulated trial was analysed using the “rjags” package in R. We assumed outcomes in each arm follows a binomial distribution, with a success probability of *p* in a sample size *X*. The logit-transformed probabilities of success for the two arms, logit(p_A_) and (logit(p_B_), were assumed to follow asymptotically normal distributions. We assigned priors to the logit function of the success probability in arm A (logit(p_A_)) and also to the difference between the two arms (log(OR)). No prior was assigned to the probability of success in arm B because it can be expressed in terms of logit(p_A_) and log(OR) [17, 18]. The choice of priors is described in the subsequent section. At every interim analysis, we assessed posterior distributions of the probabilities of success in each arm (p_A_ and p_B_) and their difference (p_B_-p_A_), with each analysis involving 10,000 iterations of Monte Carlo sampling.

### Trial design structures

**Fig. 1:**
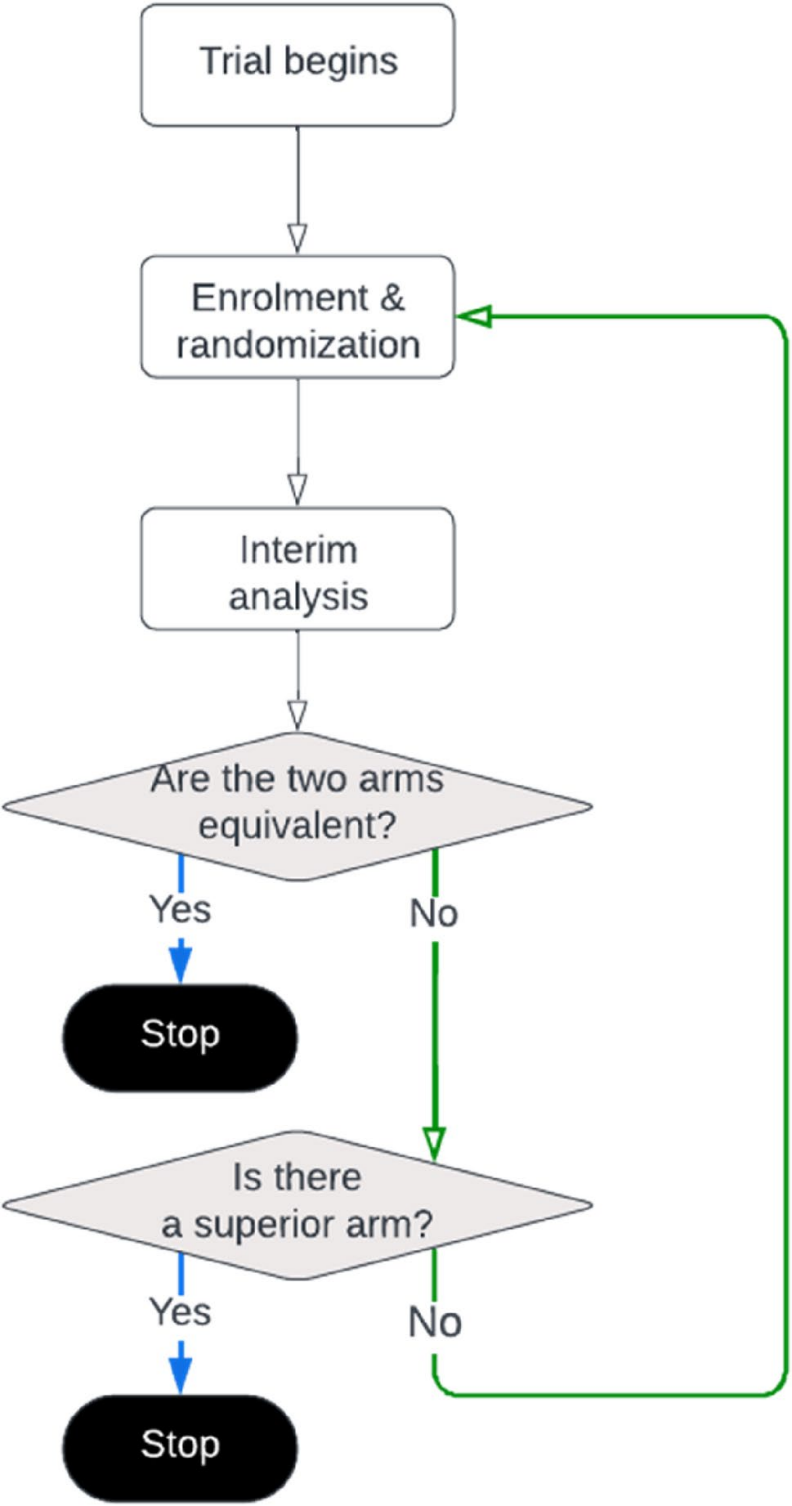
Two-arm adaptive trial design. The aim of the trial is to compare two arms, with pre-specified interim analyses allowing early termination when evidence shows that the two arms perform equally or demonstrates that the two arms perform differently, and one of the arms is clearly superior

This study used an adaptive trial design with interim analyses at predefined intervals and early stopping rules. An efficacy stopping rule was triggered if the accumulated data demonstrated that either arm showed superiority. Alternatively, an equivalence stopping rule was triggered if the two arms performed equally with a negligible effect difference. If neither stopping rules was triggered, interim analyses repeated until the maximum sample size was reached, at which point the trial outcomes were declared inconclusive (Fig. 1).

We set the maximum sample size at 100,000 to assess the performance of this trial design in a scenario where sample size is fairly unconstrained (i.e. very high-throughput, routine data). For our motivating example of the vision screening programme, about 3.6 million people were screened in the year 2024, and 20% were identified with VI. Therefore, 100,000 participants would be recruited in less than 2 months across all study sites of the programme. We also performed a supplementary analysis with a reduced sample size limit of 20,000, representing a more reasonable figure in a lower-throughput data setting or in situations where only a few study sites participate.

We explored five design features: the choice of prior distributions, the sample size at the first interim analysis, frequency of subsequent interim analyses, the threshold of the stopping rule for efficacy, and the threshold of the stopping rule for equivalence. Each feature was varied one at a time, while the others were held constant at their reference values as specified in Table 1.

**Table 1:** Trial design features

Design features	Reference	Variations
Priors	Neutral prior	Moderate sceptical prior or extreme sceptical prior
Sample size at first interim analysis	After recruiting 50 participants per arm	After recruiting 100, 200, or 500 participants per arm
Frequency of interim analysis	Every 50 extra participants per arm	Every 100 or 250 extra participants per arm
Stopping for efficacy	Stop trial if there is a high posterior probability (*S =* 95%) that one arm performs better than the other arm *Pr*(|Δeffect|> 0%) ≥ S%	$\:S\in\:(90\%,\:91\%,\:92\%,\dots\:,\:99\%)$
Stopping for equivalence	Stop trial if there is a high posterior probability (*E =* 95%) that the difference between the two arms is < 1% *Pr*(|Δeffect|< 1%) ≥ E%	$\:E\in\:(80\%,\:81\%,\:82\%,\dots\:,\:99\%)$

*S* the posterior probability threshold for stopping a trial for efficacy

*E* is the posterior probability threshold for stopping a trial for equivalence

Δ effect is the effect difference between the two arms

#### Priors

We assigned prior distributions to the probability of success in one arm (p_A_) and to the difference between the two arms. The success probability in arm A was assumed to follow a uniform distribution between 0 and 1 (p_A_ ~uniform(0,1)), which, when transformed to the logit scale, was approximated by a normal distribution (logit(p_A_)) ~ norm(0,0.3)).

We used three different prior distributions for the relative effect between arms. First, a neutral prior, centered at an OR of 1.0 with a credible interval from 1/30 to 30, allowed the posterior to be solely driven by the accumulating data [19]. Given the 50% mean success probability in arm A, this implied a rate range of 3.2% to 96.7% in arm B.

**Fig. 2:**
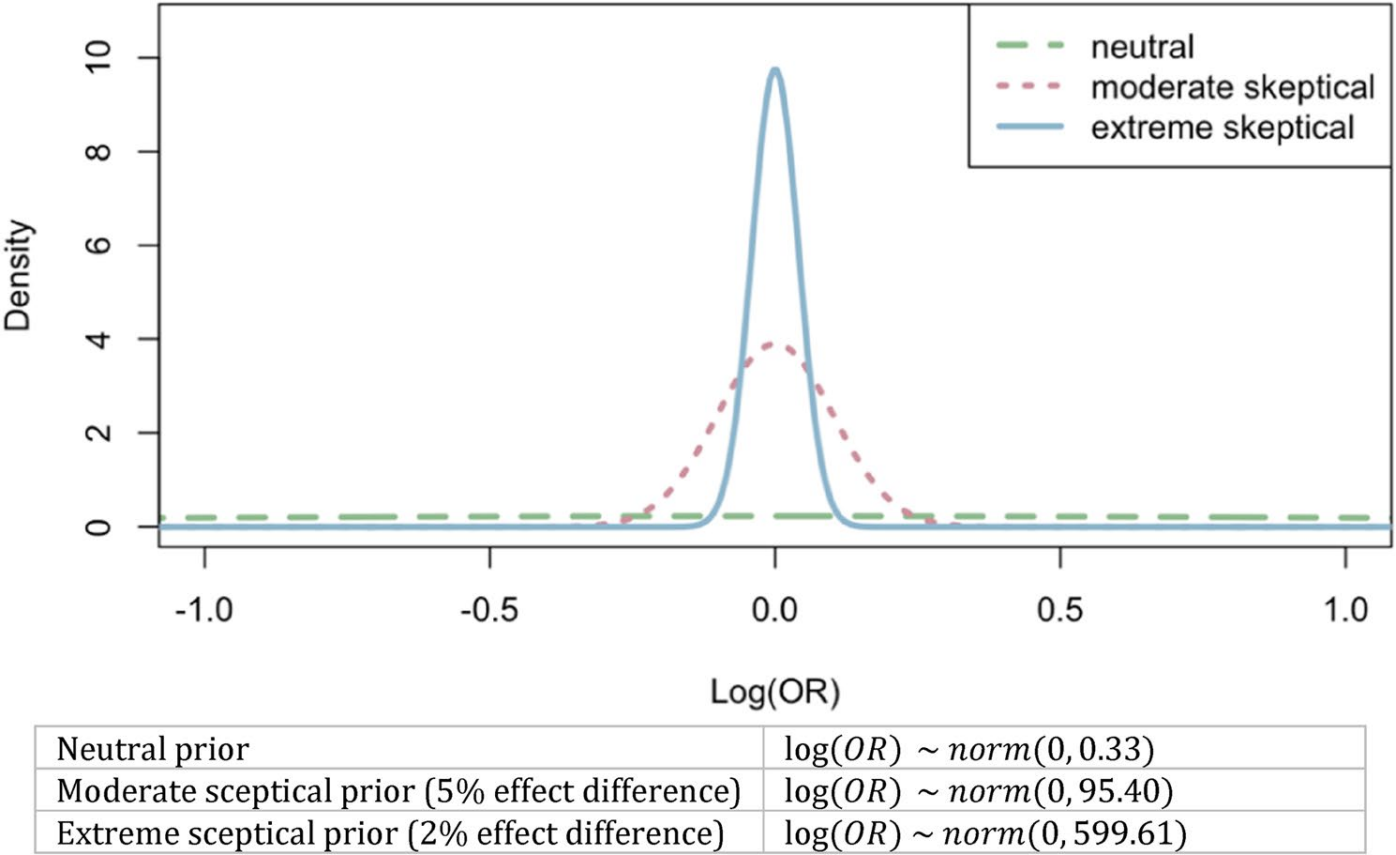
Choice of priors

Alternatively, two sceptical priors were considered to prevent overfitting of the observed data [20] and reflect the belief that programmatic changes would lead to modest improvements. The moderate sceptical prior assumed the absolute mean difference between the two arms unlikely to exceed 5% (OR 1.0; 95% CI: 0.82–1.22), and the extreme sceptical prior assumed less than 2% improvement (OR of 1.0; 95% CI: 0.92–1.08). All three priors were transformed into the log scale (log(OR)), approximated by normal distributions with a mean of 0 and corresponding precision (Fig. 2).

#### Sample size at first interim analysis

We considered conducting the first interim analysis after a prespecified run-in period. The first analysis was initiated after having outcome data available in 100, 200, 400, or 1,000 participants across both arms.

#### Frequency of interim analysis

We assessed the impact of changing the frequency of interim analyses at one of the following predefined intervals: frequent analyses every 100, moderate analyses every 200, and less frequent analyses every 500 participants across both arms.

#### Stopping for efficacy

We explored the impact of two early stopping rules: stopping for efficacy and stopping for equivalence. Trials were allowed to stop early for efficacy if one arm showed any improvement over the other with an effect difference greater than 0%. At each interim analysis, we estimated the posterior probability that the effect difference exceeded 0% Tthe trial was stopped if this probability met or exceeded a predefined efficacy threshold *S*. In this case, the arm with the higher mean probability of success was declared superior.

- If $\:P\left({(p}_{j}-{p}_{k})>0\%\:\right)\ge\:S\%$: Stop trial and declare efficacy of arm j where j and k index any two arms in a trial, and p is the success probability of an arm.

We assessed different values of the threshold *S* between 90% and 99% to determine how varying the strength of evidence required for declaring efficacy affects the trial.

#### Stopping for equivalence

Alternatively, trials were allowed to stop for equivalence if the difference between the two arms was deemed negligible. We defined equivalence using an *indifference zone* of -1% to 1%, where any difference within this range was considered small enough to declare equivalence. At each interim analysis, trials were allowed to stop if there was at least *F*% posterior probability that the effect difference falls within this zone.

- If $\:\:P\left(\left|{p}_{j}-{p}_{k}\right|<1\%\right)\ge\:E$: Stop trial and declare equivalence of the two arms j and k, where j and k index the two arms in a trial, and p is the success probability of an arm.

To assess various definitions of equivalence, we explored the impact of varying *E* from 80% to 99%.

Although the efficacy and equivalence stopping criteria are designed to be mutually exclusive, we acknowledge that both could theoretically be met if the posterior distribution of the effect difference is highly concentrated within a narrow interval (e.g., 0% to 1%). Nonetheless, we expected that such scenarios would be rare in practice due to the precision and distributional characteristics required. In the event that both criteria are met, our decision rule prioritised stopping for equivalence, reflecting a more conservative interpretation of the effect.

### Performance measures

We evaluated the following performance measures to evaluate how the trial design features perform.

#### Sample size

Sample size of a trial was recorded as the total number of participants when the trial stopped, either for efficacy, equivalence, or upon reaching the maximum sample size limit.

#### Error rates

Error rates were assessed by comparing observed trial outcomes to true outcomes in each scenario to evaluate how often correct or incorrect results occurred. The traditional definitions of type I and II error rates apply to comparing treatment arms to a control arm, but are not fully transferrable when comparing two variants of a programmatic change with minimal differences in cost and risk. When interventions are low-risk and low-cost programmatic changes focused on improving outcomes such as clinic attendance, we acknowledged that a false positive rate of wrongly declaring one variant superior has limited harm. Therefore, we adapted error rate measures that reflect the concepts of type I error and power, tailored to support programmatic decision-making in the context of the vision screening programme.

Trial outcomes were classified as *equivalence* if two arms were identified as equally effective, and *efficacy* was defined as the selection of a single superior arm. Outcomes were further categorized as *acceptable* if they led to the adoption of the truly superior arm or any of the equally performing arms. Outcomes were *less acceptable* if a superior arm is spuriously declared as equivalent to an inferior arm, potentially leading to the adoption of an inferior arm. Lastly, trial outcomes were classified *not acceptable* if an inferior arm is incorrectly identified as superior, eliminating any chance of adopting the truly superior arm (Table 2).

In scenarios where the difference between two arms lies within the region of indifference, two outcomes are possible: (a) *true equivalence*, correctly identifying both arms as equivalent; and (b) *partial efficacy*, incorrectly declaring one arm superior, similar to the type I error in frequentist trials. In this study, we acknowledged that partial efficacy, while it introduces some confusion about the truth, is still deemed acceptable because it leads to the implementation of either arm that yields comparable outcomes with no meaningful differences in cost or risk.

If two arms perform differently, three outcomes are possible: (a) *true efficacy*, correctly identifying the superior arm; (b) *false efficacy*, incorrectly declaring the inferior arm as superior; and (c) *spurious equivalence*, declaring both arms as equivalent. False efficacy and spurious equivalence combined to resemble type II error. False efficacy was considered the most unacceptable outcome as it leads to the adoption of the inferior arm, but spurious equivalence was considered less harmful, as it still left the possibility of selecting a superior arm.

**Table 2:** Trial outcomes and error rates for different scenarios

Scenarios	Trial outcome classification for programmatic decisions		
	Acceptable	Less acceptable	Not acceptable
Two arms perform equally arm A = arm B	true equivalence Two arms are declared equivalent. An acceptable result where any arm can be adopted. partial efficacy One arm is identified. An acceptable result where one arm can be adopted.	NA	NA
One arm is superior arm A < arm B	true efficacy The superior arm is identified. An acceptable result where a superior arm is adopted.	spurious equivalence Two arms are declared equivalent. A suboptimal result where there is a chance that: (a) a superior arm is adopted, or (b) an inferior arm is adopted.	false efficacy An inferior arm is identified. An error where an inferior arm is adopted.

#### Coverage and bias

Based on the evaluation of the error rates and sample sizes, we developed an optimal trial design that aligns with the study objectives and further assessed it for coverage and bias. Coverage was defined as the proportion of trials in which the 95% CIs of the posterior distributions of the observed effect difference contains the true effect difference. We estimated bias by examining both the direction and magnitude of deviation between the true and observed effect differences.

#### Probability of correctly adopting the superior arm

In the context of QI efforts, trial outcomes guide decisions on which arm to adopt into a programme. While the evaluation of error rates focuses on the statistical accuracy, programmatic decisions can still be made even when outcomes are inconclusive or show equivalence. In such cases, the variant that appears more promising based on the final data may be selected for implementation.

We considered two approaches to select an arm to implement following completion of an adaptive trial. First, under the Bayesian adaptive framework, we determined that the arm with the higher mean posterior probability of success would be adopted, including in trials that concluded equivalence or were otherwise inconclusive. This approach reflects full Bayesian inference and incorporates the influence of the specified prior. Second, we evaluated an alternative approach in which the same adaptive stopping rules were applied, but the final selection of the arm was based solely on the observed data, with the arm with the higher mean observed success provability selected. This alternative analysis approach excludes the influence of the specified prior and is intended to reflect a frequentist-style final analysis applied after adaptive stopping, where the arm with the higher mean success probability is adopted without incorporating the prior.

For comparative analysis, we simulated four sets of conventional RCTs with fixed total sample sizes of 2,000, 5,000, 10,000, or 15,000 participants, each with 1,000 simulations. The fixed sample sizes for the conventional RCT simulations were selected to reflect the typical scale of traditional clinical trials, generally ranging up to 15,000 participants or even much less. At the end of the trials, we determined that the arm with the higher success probability would be adopted, regardless of statistical significance.

For each approach, we estimated the proportion of simulated trials that correctly adopted the superior arm.

## Results

### Reference design

We created a reference trial design which has all five trial design features set to the reference values specified in Table 1, hereafter referred to as the “reference design”.

Using this reference design, all simulated trials triggered a stopping rule before reaching the sample size limit of 100,000. Trials evaluating larger effect differences had smaller sample sizes and error rates. In scenarios where one arm performed better by 1% effect difference, trials ended with a median sample size of 2,900 [IQR 500; 13,125] with an 81.0% true efficacy rate. In scenarios with a 5% effect difference, trials had a smaller median sample size of 600 [IQR 300;1,500] and a 97.3% true efficacy rate. For all scenarios with varying effect differences, most errors were false efficacy declaring an inferior arm as superior, only a few instances saw spurious equivalence, where the two arms were incorrectly declared equal (Table 3).

In scenarios with no effect difference between the arms, this reference design demonstrated a 74.5% partial efficacy rate, and only a quarter were true equivalence. This high partial efficacy rate was attributed the equivalence stopping rule being more stringent than the efficacy stopping rule, requiring large samples to conclude equivalence and led the efficacy rule to be triggered more frequently. This suggested that, if the goal is to reduce the partial efficacy rate, either the equivalence stopping rule needs to be relaxed, or the efficacy stopping rule needs to be made even stricter.

**Table 3:** Error rates and sample sizes of simulated trials using the reference design

	Winning arm identified			Sample size		
True effect difference	Arm A	Arm B	Equivalence	Median (IQR)	Minimum	Maximum
0%	38.2%	36.3%	**25.5%**	4,200 (600; 39,300)	100	89,100
1%	16.7%	**81.0%**	2.3%	2,900 (500; 13,125)	100	99,200
2%	9.5%	**90.5%**	0%	2,050 (500; 5,800)	100	47,900
3%	5.7%	**94.3%**	0%	1,100 (400; 3,025)	100	21,400
4%	3.6%	**96.4%**	0%	900 (300; 2,000)	100	14,800
5%	2.7%	**97.3%**	0%	600 (300; 1,500)	100	9,100

The first column shows the true effect difference of each scenario, and the next three columns show the trial outcomes. Values in bold indicates correct identification by the adaptive design: declaration of equivalence (true negative) when the true effect difference between arms is 0%, or correct identification of the superior arm. The last three columns show the final sample sizes across both arms overall. This reference design used a neutral prior, and interim analyses were conducted every 100 participants. Trials were allowed to stop early under two conditions: (a) stop for efficacy if there is at least a 95% posterior probability that the two arms are different by any amount; or (b) stop for equivalence if there is at least a 95% posterior probability that the effect difference between the two arms is less than 1%.

Subsequent sections assessed trial design features aimed at reducing the risk of false efficacy and spurious efficacy. Results are illustrated for scenarios with 0–3% effect differences, where design changes showed a large impact. Scenarios with 4% or 5% effect difference, where design changes had minimal influence, are presented in the appendix along with detailed error rates and sample sizes.

### Effects of prior

**Fig. 3:**
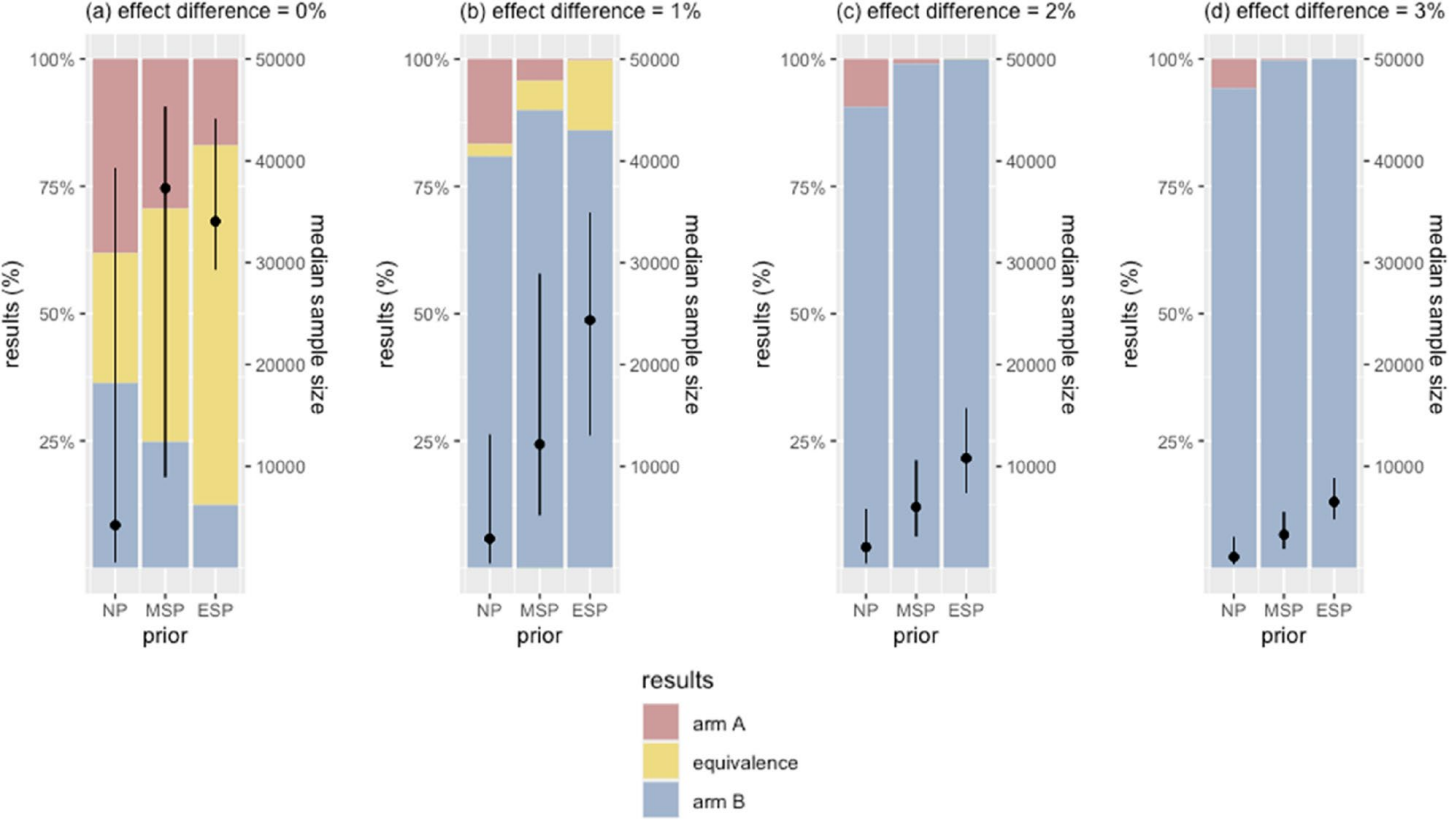
Effects of choice of priors on error rates and sample size. Bars illustrate the trial outcomes, stratified by the magnitude of the true effect difference. Black dots are the median sample sizes of simulated trials, with vertical lines indicating the interquartile ranges. X-axis shows the choice of prior distributions for effect difference, where NP is a neutral prior, MSP is a moderate sceptical prior, and ESP is an extreme sceptical prior. In figure (**a**), yellow bars represent the proportion of true equivalence outcomes that correctly concluded equivalence between the two arms. In figures (**b**)-(**d**), blue bars are the proportions of true efficacy outcomes where the superior arm was correctly identified

In scenarios with no effect difference, employing sceptical priors improved the true equivalence rate from 25.5% (neutral prior) to 45.7% (moderate sceptical prior) and to 70.5% (extreme sceptical prior) (Fig. 3 & Appendix Fig. 1).

Using the sceptical priors also improved the true efficacy rate when the two arms performed differently. In scenarios with a 1% effect difference, the true efficacy rate improved from 81.0% (neutral prior) to 86.6% (extreme sceptical prior), and to 90.0% (moderate sceptical prior). In scenarios with effect differences of 2% or greater, using the sceptical priors resulted in true efficacy rates of 99% or higher.

While sceptical priors improved accuracy, they also prolonged trial durations. In scenarios where two arms performed equally, using the neutral prior had a median sample size of 4,200 [IQR 600; 39,300], but the moderate sceptical prior and the extreme sceptical prior increased the median sample sizes to 37,300 [IQR 8,900; 45,350] and 34,050 [29,300 − 44,100], respectively. This increase occurred because, in trials that declared partial efficacy, more evidence was needed to overcome the prior belief strongly centred around no effect difference. 

In contrast, sample sizes for trials that correctly concluded equivalence remained relatively consistent across priors, with the extreme sceptical prior requiring slightly fewer participants.

In scenarios where one arm performed better, using sceptical priors required stronger evidence to counter the belief of no effect difference. In scenarios with a 1% effect difference, using the neutral prior resulted in a median sample size of 2,900 [IQR 500; 13,150], while the moderate and extreme sceptical priors raised the sample sizes to 12,150 [IQR 5,200; 28,925] and 24,350 [IQR 13,075 − 34,900], respectively. This increase in sample size was consistently observed in other scenarios with larger effect differences.

Overall, sceptical priors were advantageous for reducing the false efficacy rates, especially when the true effect difference between the two arms is small. However, this benefit came at the cost of markedly larger sample sizes, in some scenarios several times greater than those required with a neutral prior.

### Effects of sample size at the first interim analysis

**Fig. 4:**
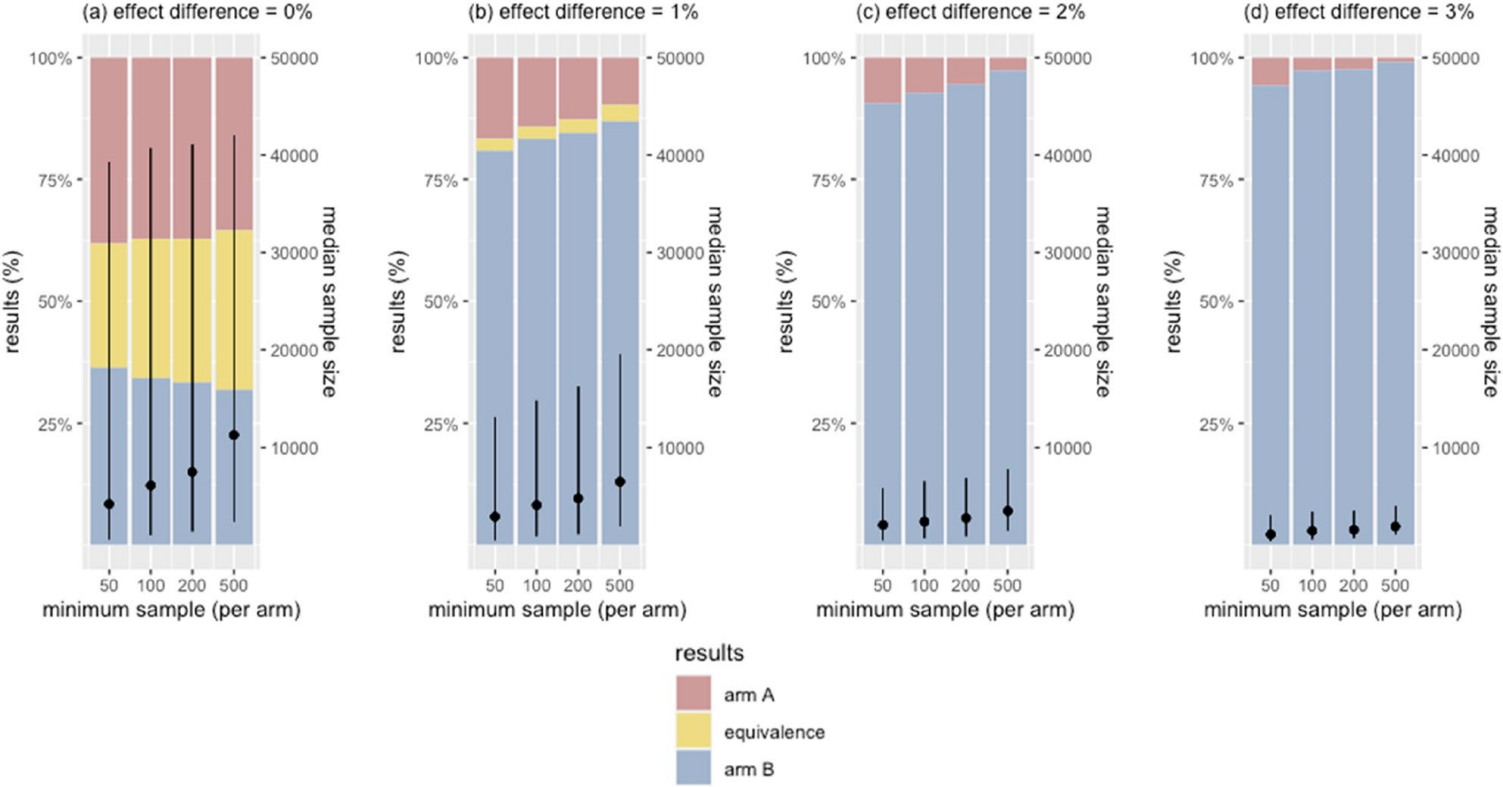
Effects of sample size at first interim analysis on error rates and sample size. Bars illustrate the trial outcomes, stratified by the magnitude of the true effect difference. Black dots are the median sample sizes of simulated trials, with vertical lines indicating the interquartile ranges. X-axis shows the sample size accrued before conducting the first interim analysis. In figure (**a**), yellow bars represent the proportion of true equivalence outcomes that correctly concluded equivalence between the two arms. In figures (**b**)-(**d**), blue bars are the proportions of true efficacy outcomes where the superior arm was correctly identified

We varied the number of observations needed at the first interim analysis: 50, 100, 200, or 500 participants per arm. Accruing larger samples made the posterior distributions more reflective of the true effect difference, reducing the overall error rates. However, this was offset by larger final sample sizes (Fig. 4 & Appendix Fig. 2).

In scenarios where two arms performed equally, the partial efficacy rate was 74.5% with 50 participants per arm and decreased to 67.5% with 500 per arm. In scenarios with an effect difference between the arms, the false efficacy rate also went down by accruing larger samples before the first interim analysis.

### Effects of frequency of analysis

**Fig. 5:**
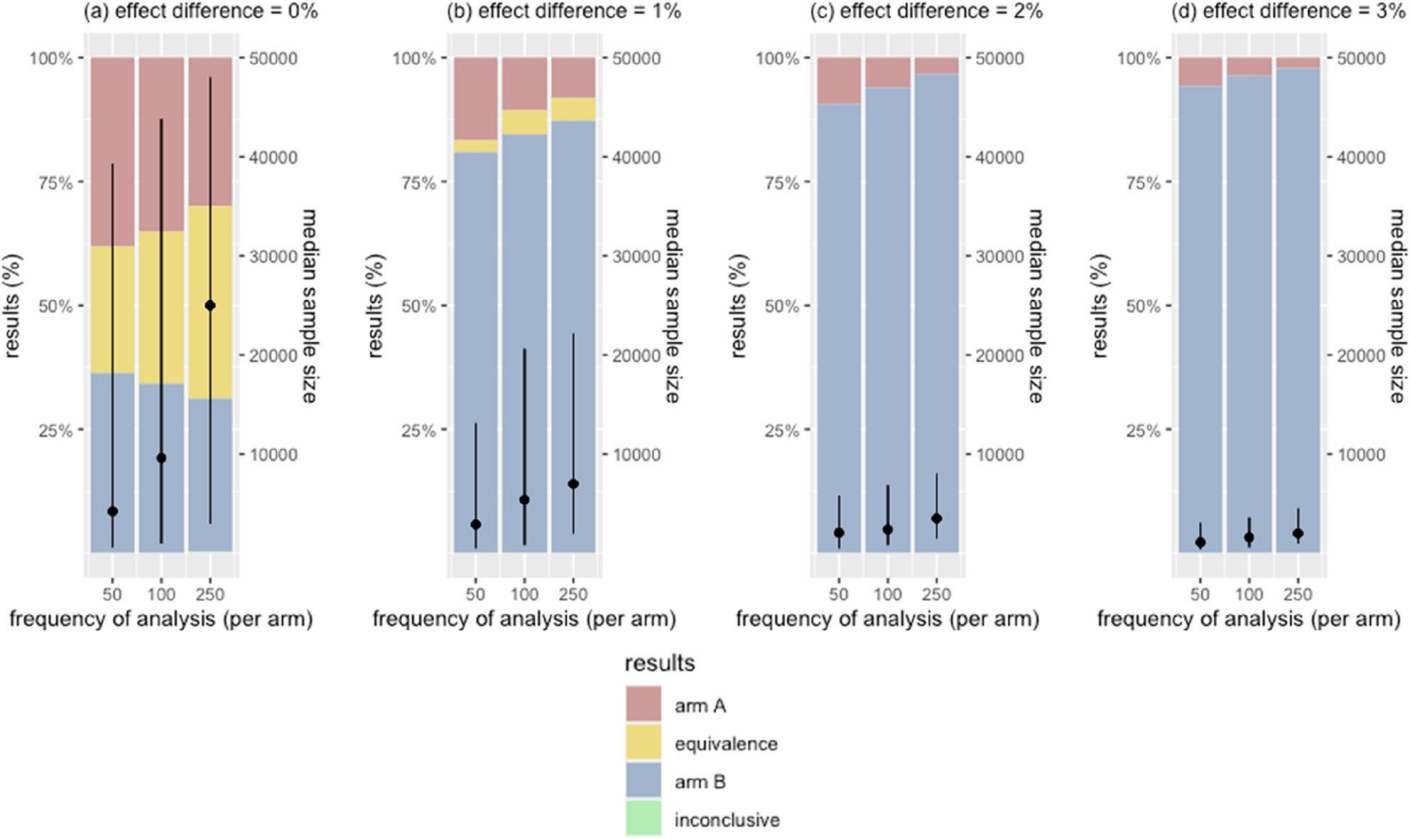
Effects of frequency of analysis on error rates and sample size. Bars illustrate the trial outcomes, stratified by the magnitude of the true effect difference. Black dots are the median sample sizes of simulated trials, with vertical lines indicating the interquartile ranges. X-axis shows the number of extra observations for interim analyses. In figure (**a**), yellow bars represent the proportion of true equivalence outcomes that correctly concluded equivalence between the two arms. In figures (**b**)-(**d**), blue bars are the proportions of true efficacy outcomes where the superior arm was correctly identified. Green bars indicate trials that hit the maximum sample size before drawing a conclusion

In scenarios where two arms performed equally, reducing the frequency of interim analysis improved the true equivalence rate from 25.5% (every 50 per arm) to 30.7% (every 100 per arm) and to 38.5% (every 250 per arm). In scenarios with a 1% effect difference, the true efficacy rate improved from 81.0% (every 50 per arm) to 84.7% (every 100 per arm) and to 87.2% (every 250 per arm) (Fig. 5 & Appendix Fig. 3).

While less frequent interim analyses increased accuracy, it also prolonged the overall trial durations. In scenarios with no difference, interim analyses every 50 per arm resulted in a median sample size of 4,200 [IQR 600; 39,300]. Conducting analyses every 100 per arm increased the median sample sizes to 9,600 (IQR 1,000; 43,800), and every 250 per arm increased it further to 25,000 (IQR 3,000; 48,000). The increasing trends were observed in other scenarios where the two arms performed differently.

### Effects of stopping rule for efficacy

We explored the impact of efficacy stopping rule (*Pr*(|Δeffect|> 0%) ≥ S%) by varying the posterior probability threshold to declare efficacy (*S*) between 90% and 99%. Across all scenarios, using a higher, more stringent threshold *S* improved accuracy. In scenarios where two arms performed equally, raising *S* from 95% to 99% reduced the risk of premature stopping for partial efficacy and allowed more data to accrue, improving the true equivalence rate from 25.5% to 66.6%. In scenarios where one arm performed 1% better, the true efficacy rate from 81.0% to 86.4%. In scenarios with 3% or larger effect differences, the true efficacy rate reached up to 99% with a higher *S*.

**Fig. 6:**
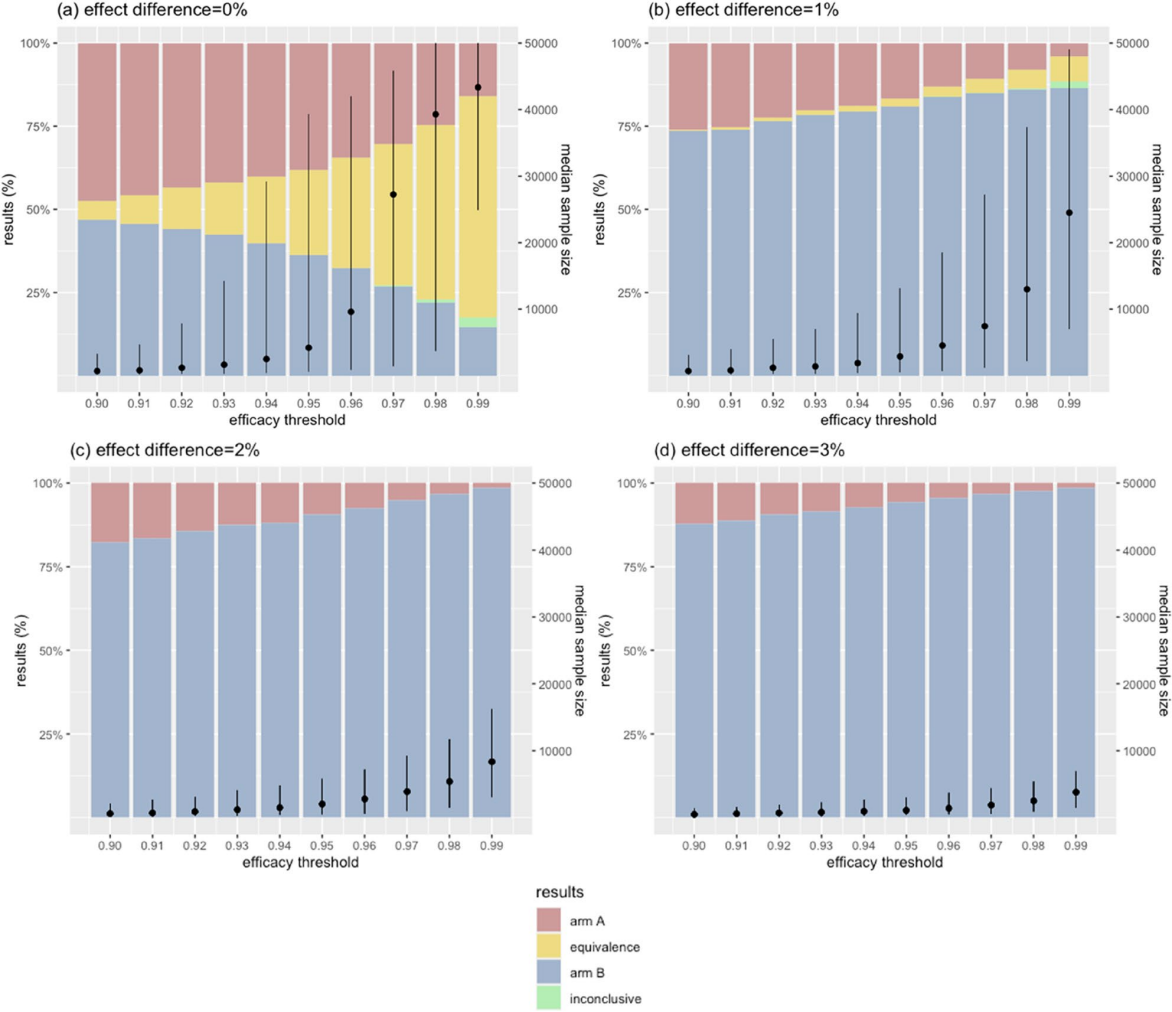
Effects of efficacy threshold on error rates and sample size. Bars illustrate the trial outcomes, stratified by the magnitude of the true effect difference. Black dots are the median sample sizes of simulated trials, with vertical lines indicating the interquartile ranges. X-axis shows the threshold values used to stop trials early for efficacy. In figure (**a**), yellow bars represent the proportion of true equivalence outcomes that correctly concluded equivalence between the two arms. In figures (**b**)-(**d**), blue bars are the proportions of true efficacy outcomes where the superior arm was correctly identified. Green bars indicate trials that hit the maximum sample size before drawing a conclusion

However, applying a more stringent efficacy stopping rule also reduced the chance of early stopping. In scenarios with 0% or 1% effect difference, using *S* of 99% led 2.9% and 2.0% of all trials, respectively, to reach the sample limit of 100,000 without stopping. This increased the average trial durations compared to designs using lower *S* to declare efficacy (Fig. 6).

### Effects of stopping rule for equivalence

**Fig. 7:**
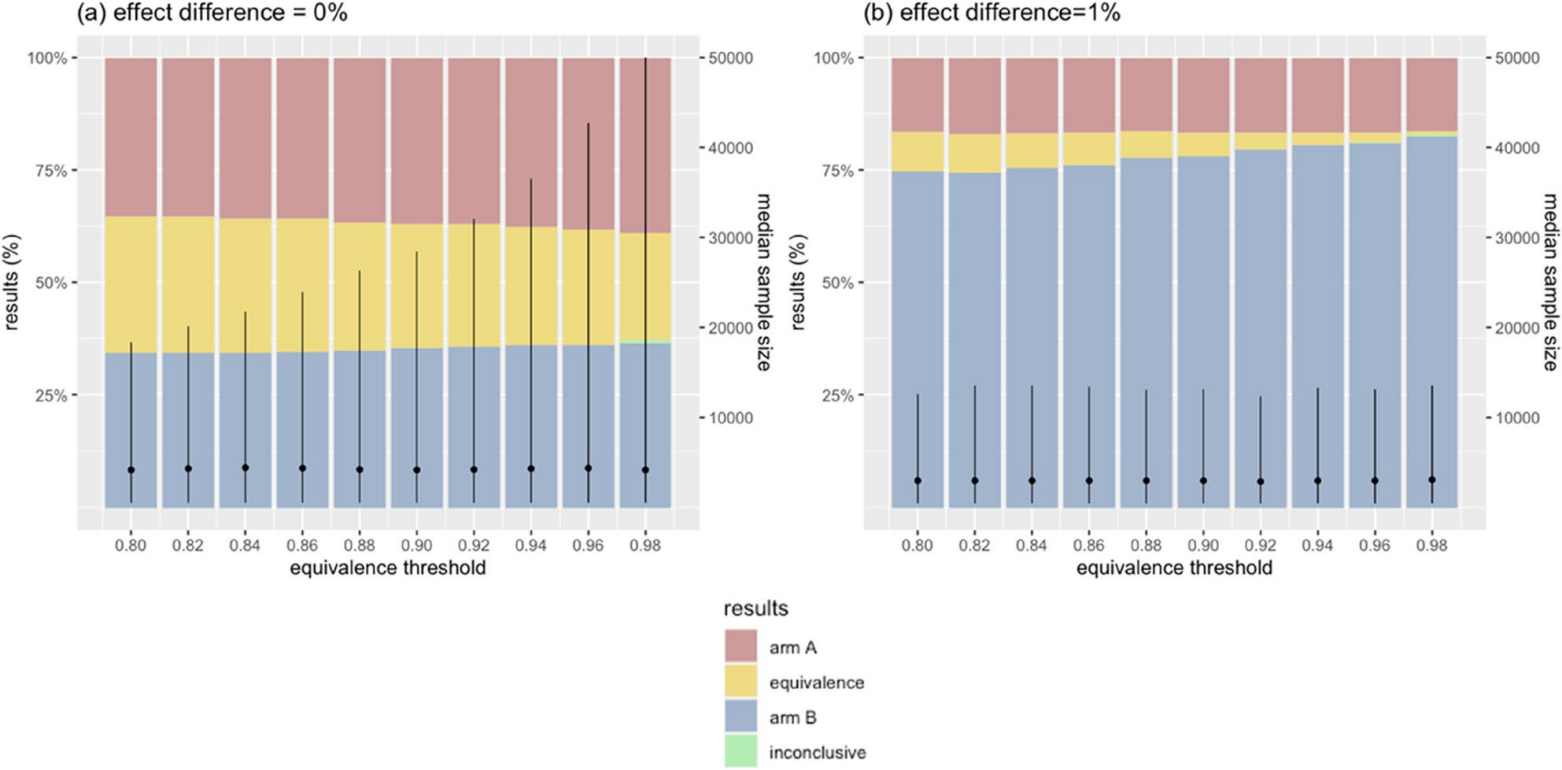
Effects of equivalence threshold on error rates and sample size. Bars illustrate the trial outcomes, stratified by the magnitude of the true effect difference. Black dots are the median sample sizes of simulated trials, with vertical lines indicating the interquartile ranges. X-axis shows the threshold values used to stop trials early for equivalence. In figure (**a**), yellow bars represent the proportion of true equivalence outcomes that correctly concluded equivalence between the two arms. In figures (**b**), blue bars are the proportions of true efficacy outcomes where the superior arm was correctly identified. Green bars indicate trials that hit the maximum sample size before drawing a conclusion

Using the reference design, which incorporated an equivalence stopping rule with E = 95% (*Pr*(|Δeffect|< 1%) ≥ 95%), trials rarely stopped for equivalence before either stopping for efficacy or reaching the sample size limit. This suggested that the equivalence stopping rule was stringent, potentially preventing trials from ending early when the two arms are truly equivalent. Therefore, we assessed the impact of a broad range *E* between 80% and 99% (Fig. 7 & Appendix Fig. 5).

Relaxing the stopping rule with a low *E* led more trials to stop for equivalence, increasing both true and spurious equivalence rates. In scenarios where two arms perform equally, lowering the equivalence threshold *E* from 99% to 80% improved the true equivalence rate from 21.3% to 30.3%. Although this increase in accuracy was relatively modest, it was achieved with notably smaller sample sizes (4,500 [IQR 600; 64,150] vs. 4,200 [IQR 600; 18,325], respectively), preventing trials from running unnecessarily long.

In scenarios with a 1% effect difference, relaxing *E* from 99% to 80% increased the spurious equivalence rate from 0.1% to 8.7%, which in turn reduced the true efficacy rate from 82.8% to 74.7% while maintaining similar sample sizes. In other scenarios with effect differences of 2% or greater, changing the equivalence stopping rule had no impact on accuracy or sample size, because all trials declared efficacy and none declared equivalence, regardless of *E* value.

### Configuring the trial design

Based on the findings, we identified two design features with notable impact on trial performance: sceptical priors which improved accuracy, and relaxed equivalence stopping rule that reduced sample size. Combining different levels of these design features simultaneously, we developed four candidate designs to propose an optimal trial design for comparing two arms with small effect differences, under the assumption of negligible cost and no associated harms – making either arm acceptable when no true difference exists. The primary focus was to identify a trial design that improved the true efficacy rate for detecting a superior arm with even modest improvements, while enabling rapid identification with practical sample size requirements. In all candidate designs, we fixed the efficacy threshold *S* at 95%, as deviations from this value showed a noticeable increase in either error rates or sample size. The reference design in Table 3 was included for comparison (Table 4).

**Table 4:** Candidate trial designs for optimizing trial design

Trial design	Prior	Initial sample size *N*	Frequency *N*	Efficacy threshold %	Equivalence threshold %
Reference	Neutral	100	100	95	95
I	Neutral	100	100	95	80
II	Moderate sceptical	100	100	95	80
III	Extreme sceptical	100	100	95	80

**Fig. 8:**
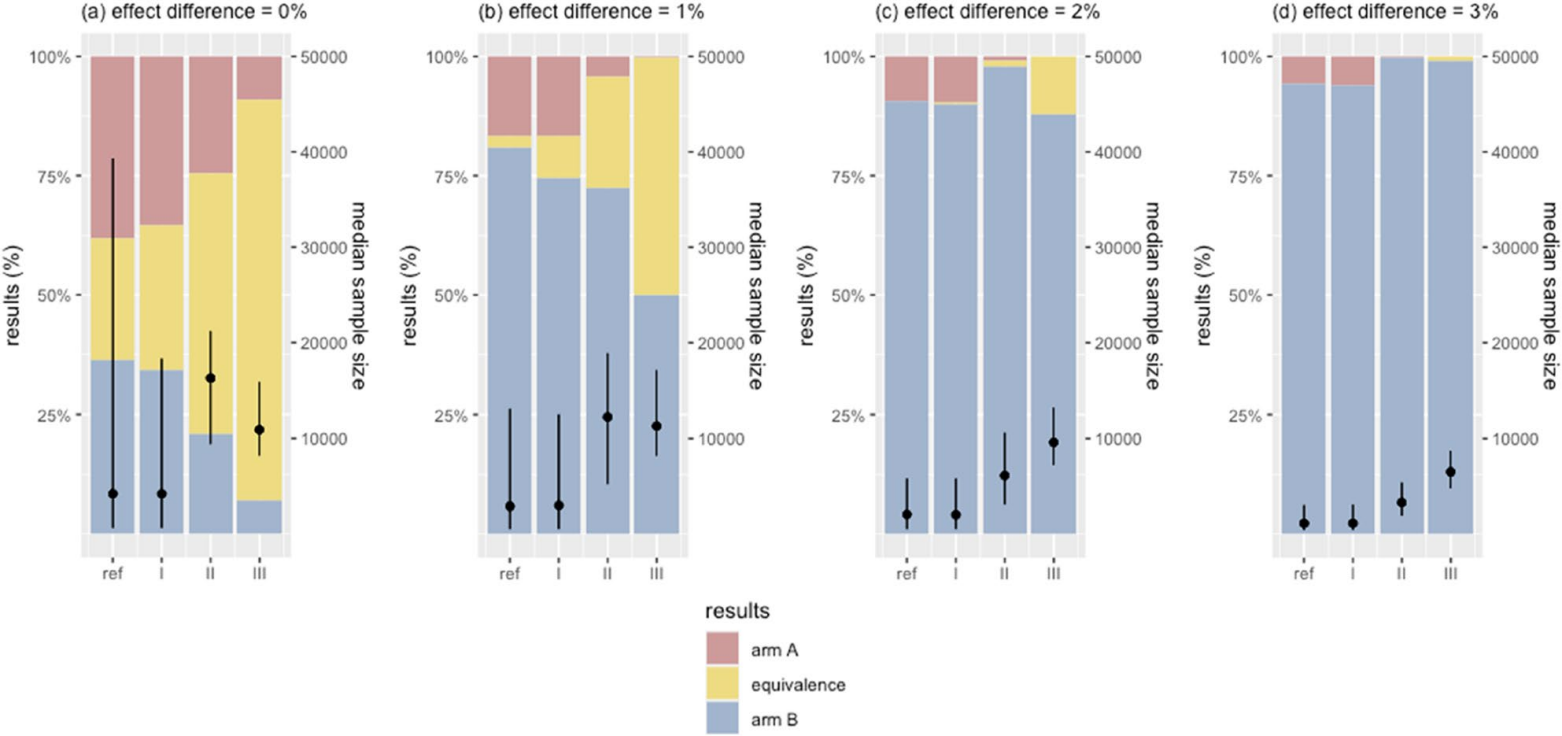
Effects of trial design on error rates and sample size. Bars illustrate the trial outcomes, stratified by the magnitude of the true effect difference. Black dots are the median sample sizes of simulated trials, with vertical lines indicating the interquartile ranges. In figure (**a**), yellow bars represent the proportion of true equivalence outcomes that correctly concluded equivalence between the two arms. In figures (**b**)-(**d**), blue bars are the proportions of true efficacy outcomes where the superior arm was correctly identified

Figure 8 and Appendix Fig. 6 illustrate the performance of these four trial designs with the maximum sample size limit set to 100,000. The supplementary analysis presents the analysis of these trial designs repeated with a reduced sample size limit of 20,000.

In scenarios where two arms perform equally, the combination of a sceptical prior and a relaxed equivalence stopping rule effectively improved the true equivalence rate. Design (I) with the neutral prior had a true equivalence rate of 30.3%, while design (III) with the extreme sceptical prior had a substantially higher rate of 84.1%. Design (I) had the smallest median sample size of 4,200 [IQR 600; 18,325] (design (I)), followed by design (III) (10,900 [IQR 8,200; 15,900] and design (II) (16,300 [IQR 9,400; 21,200]).

However, improving the true equivalence rate came at the cost of a reduced true efficacy rate, leading more trials failing to correctly identify the superior arm. In scenarios with a 1% effect difference, the reference design had the highest true efficacy rate at 81.1%. Introducing sceptical priors and relaxed equivalence stopping rule reduced this rate to 72.6% (design (II)) and to 50.1% (design (III)). But in these designs, most trials that failed to conclude true efficacy were spurious equivalence rather than false efficacy – an important distinction, as false efficacy were more concerning for QI decision-making. In contrast, designs using a neutral prior (reference design and design (I)) produced higher false efficacy rates, putting a greater risk of adopting an inferior arm.

In scenarios with a 2% effect difference, all three candidate designs showed comparable or higher true efficacy rate than the reference design. Again, most of the errors in trials using sceptical priors (designs (II) and (III)) were spurious equivalence outcomes, and the risk of false efficacy outcomes was minimal. Design (II) had the highest true efficacy rate of 97.8% compared to 90.8% using the reference design, but at the cost of a larger median sample size (6,100 [IQR 3,100; 10,600] vs. 2,050 [IQR 500; 5,800]). In scenarios with effect differences of 3% or greater, using sceptical priors (designs (II) and (III)) showed high true efficacy rates approaching 100%.

#### Error rates and sample sizes of the selected design

Based on these results, design (II) emerged as an optimal design for the vision screening programme. With a sample size limit of 100,000, it showed a 54.8% rate of correctly concluding two arms as equally performing with a median sample size of 16,300 [IQR 9,400; 21,200]. When one arm was 1%, 2%, or 3% better than the other, design (II) correctly identified the superior arm with true efficacy rates of 72.6%, 97.8%, and nearly 100%, respectively. While the true efficacy rate was 72.6% with a 1% effect difference, 23.2% of trials resulted in spurious equivalence, and only 4.2% incorrectly identified the inferior arm as superior. With larger effect differences, false efficacy rates dropped further to just 0.9% when the inferior arm was 2% worse, and 0.2% when it was 3% worse. As stated above, our priority was to minimize these false efficacy rates, even if it involves accepting some spurious equivalence outcomes.

These results also demonstrate variation in required sample size under the adaptive design according to the magnitude of the true effect difference. Design (II) required a median final sample size of approximately 16,000 participants when the effect difference was as small as 1%, but only about 2,000 when the difference was 5%. In contrast, fixed-size RCTs require the total sample size to be prespecified based on an assumed effect size and proceed to this target without adaptation to the accumulating data. These trials would continue enrolling participants when the observed effect is large and clear early on, or conversely, may fail to correctly detect small effects if the prespecified sample size is insufficient.

#### Coverage of the selected design

To evaluate coverage in this trial design, we assessed whether the final 95% CIs of the posterior distributions captured the true effect differences. It is important to note that this coverage is a frequentist concept and is not directly analogous to Bayesian designs, which utilises the posterior probability of parameter values. In scenarios where the two arms perform equally, 99.6% of trials included 0% difference within their 95% CIs, with CIs narrowing around 0% as sample size increased. In scenarios with a 1% effect difference, 95.0% of trials captured the true effect difference, and coverage dropped to 90.5% for a 5% effect difference. This decline reflects the influence of the sceptical prior, which pulled estimates toward 0% effect difference, leading to underestimation especially when the true effect difference was larger. While the lower coverage rate may be less relevant in the Bayesian context, it was acceptable in this study because the posterior distributions still favoured the true superior arm, so that the superior arm could still be correctly identified.

#### Bias of the selected design

We also assessed the deviation of the observed effect difference from the true value. In scenarios where two arms performed equally, the observed mean of the posterior distribution of all trials were symmetrically distributed around the 0% true difference, with those trials that ended with smaller sample sizes more likely to deviate away from this true value. In fact, using this trial design, all trials that correctly declared equivalence had sample sizes larger than 14,000, while trials that ended with sample sizes smaller than 14,000 all concluded partial efficacy.

Due to the sceptical prior in the trial design, the observed effect differences were more likely to be underestimated when the true effect difference was larger. Nonetheless, the direction of these underestimated effects still aligned with the true difference, allowing for the correct identification of the superior arm. Therefore, despite the risk of underestimating large effect differences, this bias was deemed acceptable within the context of our study, where the primary objective was to quickly identify the superior arm that perform better by any effect size.

To examine the influence of the sceptical prior on effect estimation, we repeated the final analysis in which trials followed the same adaptive stopping rules but the final effect difference was estimated using a frequentist-style approach based solely on the accumulated data, without incorporating the specified prior. This allowed us to separate the impact of early stopping rules from the impact of the prior on the observed effect estimates. It showed that, in the absence of the sceptical prior in the estimation, the observed effect was likely to be overestimated due to the effect of early stopping. The positive bias was most pronounced in scenarios with marginal effect differences, such as 1%, where trials were more likely to stop early for efficacy based on an initially inflated observed effect (Appendix Fig. 7).

#### Probability of correctly adopting the superior arm

**Fig. 9:**
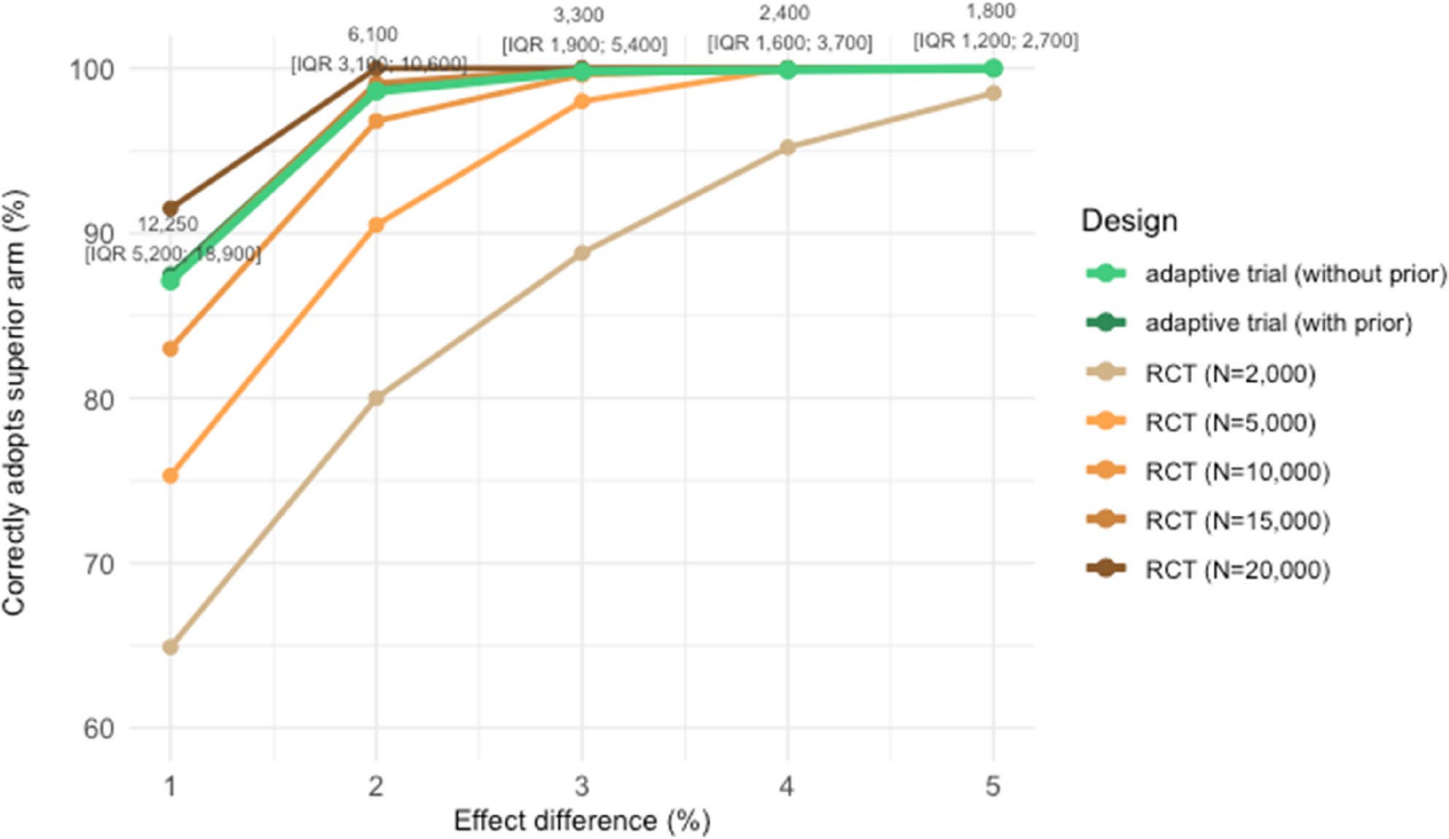
Probability of correctly adopting superior arm in adaptive trial and fixed-size RCT. The x-axis shows the true effect difference between two arms, and the y-axis shows the probability of correctly adopting the superior arm using different trial designs. The green lines show the probabilities in the adaptive trial design with or without the incorporation of sceptical prior. The labels indicate the median and interquartile range of the sample sizes of these adaptive trials. The orange lines show the probabilities in fixed-size RCTs

To assess the practical decision-making needs in QI, Fig. 9 compares the probability of correctly adopting the superior arm between this selected design and fixed-size RCTs. Overall, our adaptive trial design demonstrated comparably high probability of correctly adopting the superior arm as RCTs but with considerably smaller sample sizes.

When one arm performed 1% better than the other arm, this adaptive trial design – with a median sample size of 12,250 [IQR 5,200; 18,900] – showed an 87.5% and 87.1% probability of correctly adopting this superior arm, with and without incorporating a sceptical prior, respectively. In contrast, this probability was achieved using RCTs that had a fixed sample size of at least 15,000.

When one arm performed 2% better, adaptive trials had more than 98.5% probability in correctly adopting the superior arm with a median sample size of 6,100 [IQR 3,100; 10,600]. This probability was only achievable in RCTs enrolling more than 10,000 participants. When one arm was 3% better, adaptive trials correctly adopted this superior arm 99.8% of the time with a median sample size of 3,300 [IQR 1,900; 5,400]. This was achievable using RCTs with a fixed sample size of more than 5,000.

## Discussion

This study explored the use of adaptive trial designs for comparing two variants of a programmatic change for QI. As recommended by the U.S. Food and Drug Administration (FDA) [21, 22], simulations were used to assess the performance of various adaptive trial designs across plausible scenarios in which the two variants had small effect differences. We used these results to identify trial design features that offer a high probability of correctly adopting the variant that yields even a marginal improvement, while maintaining sample size requirements within practical limits for the vision screening programme and similar high-throughput, routine data settings.

In this study, our priority was to minimize the false efficacy rate of mistakenly identifying an inferior variant as superior. Incorporating early stopping rules into the trial design achieved a higher probability of correctly adopting the superior arm with substantially smaller sample sizes than fixed-size RCTs. This efficiency was most noticeable when two variants had marginal differences in effect – a scenario that typically demands larger samples in fixed-size RCTs. Using this adaptive trial design, there was a 4.2% chance that an inferior variant would be mistakenly adopted when it was actually 1% less effective. In comparison, a conventional, fixed-size RCT with a similar sample size of 15,000 had a 12.7% risk. Even when RCTs had a large sample size of 20,000, there was still an 8.4% chance in incorrectly adopting the inferior variant. When the superior variant was 2% more effective, adaptive trials – which had a median sample size of 6,100 (IQR 3,100; 10,600) – showed only a 0.9% risk of incorrectly adopting the inferior variant, compared to a fixed-size RCT with sample size of 15,000 that had a 0.8% risk. The gains in correctly identifying a superior variant were traded off against reduced accuracy when the two variants performed equally. This trial design had higher-than-desired partial efficacy rates, where either variant was declared superior 45.2% times when, in fact, there was no discernible difference between them. While adjusting some trial design features could potentially reduce this partial efficacy rate, it required a substantial increase in sample sizes for only a slight reduction in the error rate. For example, conducting more frequent interim analysis reduced the overall sample size but increased the chance of partial efficacy outcomes. This was consistent with previous studies that demonstrated the effect of more frequent interim analysis, coupled with an early stopping rule for efficacy, on inflating the type I error [23]. However, we assumed a study setting where two variants had comparable implementation costs and minimal safety concerns. Therefore, the potential harm of occasionally selecting one of the two equivalent variants as superior was expected to be minimal. A tradeoff was made, accepting lower accuracy when the two variants perform equally, in exchange for achieving higher accuracy if even a small improvement is observed. In settings where the two variants have different implementation costs or risks, then an alternative set of specifications for the trial is needed, as this could increase the harm of incorrectly concluding efficacy. Previously studies have emphasized the importance of controlling for bias, especially when detecting small improvements because distinguishing small effects from the null can be tricky [24]. Due to the use of early stopping rules and a sceptical prior, this adaptive trial design was prone to bias in the estimated effect difference. Early stopping and a sceptical prior had opposing effects, where early stopping for efficacy tended to inflate the estimated effect [25, 26], and the sceptical prior pulled estimate towards the null. When the true effect was small as 1%, the observe effect was generally overestimated, driven by the impact of early stopping rules. But when the true effect was large, the observed effect was underestimated because the sceptical prior exerted a stronger pull towards the null. The biased estimates should be interpreted with caution, especially to avoid poor decision-making for QI efforts. Nonetheless, in this study, the bias generally favoured the truly superior variant, which means that the trial’s ability to correctly identify the superior variant was unaffected. We reiterate that this study focused on quickly identifying a superior variant with any improvement, rather than quantifying the exact amount of that improvement, which helped overcome the limitations imposed by bias.

There are several suggestions for those interested in adopting this two-arm adaptive trial design for QI efforts. First, this study demonstrated that using sceptical priors could improve the chance of correctly identifying a superior variant when the two variants had small effect differences. However, this benefit came at the cost of markedly larger sampler sizes, sometime several times larger than those applying a neutral prior instead. This should be carefully considered given that sample size reduction through early stopping is a significant motivation for adopting adaptive designs. When selecting a prior, we recommend carefully selecting a distribution that best reflects the expected effect differences, which can be based on expert knowledge or evidence from similar settings – practices commonly used in current QI efforts [27].While the large sample sizes presented in this study are feasible for the high-throughput setting of our vision screening programme, they may be considered too large and not feasible within many other programmatic settings. Factors such as challenges of recruitment and loss to follow-up were not accounted for, which could potentially lead to different sample size requirements in pragmatic settings. In that case, a more manageable sample size limit can be set as an additional stopping criterion. If no superior variant is identified by the time this limit is reached, researchers may consider concluding that there is insufficient evidence of an improvement and move onto evaluating other programmatic changes. Our supplementary analysis showed that lowering the sample size limit increases the risk of false equivalence, often resulting in a failure to identify the superior variant, especially when the magnitude of the improvement is small.

While adaptive trial designs with lower maximum sample size limits have limitations, fixed size designs also have notable drawbacks. Fixed sample size designs require enrolling the full predetermine number of participants before drawing conclusions, potentially delaying decision-making and prolonging exposure to less effective variants. Therefore, in programmatic settings where timely decisions and resource conservation are critical, adaptive trial designs present a valuable approach to improve efficiency and flexibility.

Several limitations of this study point to potential areas for future research. First, a theoretical edge case exists in which both efficacy and equivalence stopping criteria could be satisfied simultaneously, particularly in very large trials with extremely narrow posterior distributions centred near the boundaries between the two stopping rules. Although this scenario was not observed in any of our simulations, we have pre-specified that equivalence would take precedence in such cases. This choice reflects a more conservative interpretation of the evidence and supports safer decision-making in borderline scenarios.

Second, while this study focused on a low-risk, programmatic setting, we acknowledge that many existing evaluations of adaptive trial designs adopt conventional definitions of type I and II error, particularly in clinical trials involving interventions with potential harms. More consequential QI initiatives, such as those targeting clinical outcomes like morbidity or mortality, may warrant stricter control of error rates and more conservative decision rules. In contrast, our adapted definitions were designed to better reflect the practical consequences of decisions in the context of a vision screening programme, where both variants carried minimal risk and cost differences. Nonetheless, future work could benefit from comparing standard and context-specific error definitions to clarify how various design features perform across different trial contexts.

Third, our approach to design exploration involved clarifying the impact of each design feature on trial performance. However, we recognise that this method can oversimplify the design space and may obscure important interactions between parameters. In applied settings, a more thorough evaluation of parameter combinations is needed to identify truly optimal designs tailored to the specific goals and constraints of the intended QI setting.

## Conclusions

This adaptive trial design shows promise for QI efforts aiming to compare two variants of a programmatic change and identify the one that yields a small improvement. It provides an alternative approach to both RCTs and quasi-experimental methods of selecting variants, increasing the accuracy in detecting small improvements with relatively small sample sizes.

## Supplementary Information

Supplementary Material 1.

Supplementary Material 2.

## Data Availability

The codes to generate and analyze datasets for this study are available at: (https://github.com/mjkim29/imseen).

## Abbreviations

CI: Credible interval
FDA: U.S. Food and drug administration
IQR: Interquartile range
OR: Odds ratio
QI: Quality improvement
RCT: Randomised controlled trial
VI: Vision impairment
