# Supplementary material for: Bayesian adaptive trial designs for evaluating low-risk programmatic changes for quality improvement in health services: a simulation study
Source: BMC Med Res Methodol. 2026 Feb 27;26:75. doi: 10.1186/s12874-026-02780-w (PMC13049811; doi:10.1186/s12874-026-02780-w)
Supplement: Supplementary file 1 — Supplementary Material 1. [file 12874_2026_2780_MOESM1_ESM.docx]

**Title:**

Bayesian adaptive trial designs for data-driven quality improvement in health services: a simulation study

**Journal:**

BMC Medical Research Methodology

**Authors:**

Min Jung Kim^1^, David Prieto-Merino^1,2^, Jennifer Nicholas^1^, Luke Allen^1^, Andrew Bastawrous^3^ , David Macleod^1^

^1^ Faculty of Epidemiology and Population Health, London School of Hygiene & Tropical Medicine, London, UK

^2^ Universidad de Alcalá, Madrid, Spain

^3^ International Centre for Eye Health, Clinical Research Department, London School of Hygiene & Tropical Medicine (LSHTM), London, United Kingdom.

Author information

| **Author** | **Email address** |
| --- | --- |
| Min Jung Kim | min.kim@lshtm.ac.uk |
| David Prieto-Merino | david.prieto@lshtm.ac.uk |
| Jennifer Nicholas | jennifer.nicholas@lshtm.ac.uk |
| Luke Allen | luke.allen@lshtm.ac.uk |
| Andrew Bastawrous | andrew.bastawrous@lshtm.ac.uk |
| David Macleod | david.macleod@lshtm.ac.uk |

Corresponding author

| **Name** | Min Jung Kim |
| --- | --- |
| **Email address** | min.kim@lshtm.ac.uk |
| **Address** | London School of Hygiene and Tropical Medicine  Keppel Street, London, United Kingdom WC1E 7HT |

#### Performance of the candidate trial designs with a maximum sample size limit of 20,000

By putting a smaller sample size cap, trial designs that typically required large sample sizes before stopping were more likely to yield inconclusive outcomes instead. This was particularly evident in scenarios with effect differences of 2% less. In scenarios with effect differences of 3% or greater, all trials ended with before reaching the limit of 20,000, and neither the error rates nor sample sizes were affected.

In scenarios with no effect difference, a large proportion of trials could not stop for equivalence before reaching the sample size limit, leading to inconclusive outcomes. For example, in design (II), the likelihood of inconclusive outcomes was 28.9%. However, if inconclusive outcomes were treated as equivalence for decision-making purposes, design (II) would still achieve an overall equivalence rate of 61.7%. This is in fact higher than the 54.8% true equivalence rate observed when the sample size limit was set to 100,000.

In scenarios with a 1% effect difference, design (II) also produced the highest proportion of inconclusive outcomes of 22.1%. When combined with the 14.0% spurious equivalence rate, this resulted in an overall spurious equivalence rate of 36.1%. Therefore, lowering the sample size limit from 100,000 to 20,000 increased the risk of observing spurious equivalence outcomes from 27.4% to 36.1%, and the true efficacy rate decreased from 72.6% to 59.8. Similarly, in scenarios with a 2% effect difference, the true efficacy rate decreased from 97.8 % to 87.9%. This indicated that lowering the sample size limit reduces the true efficacy rate of identifying the superior arm when the effect difference is as small as 1% or 2%. In scenarios effect differences of 3% or greater, lowering the sample size limit to 20,000 had little impact because all designs already showed true efficacy rates close to 100%.

***Supplementary figure 1****. Effects of trial design on error rates and sample size* with a maximum sample size limit of 20,000.


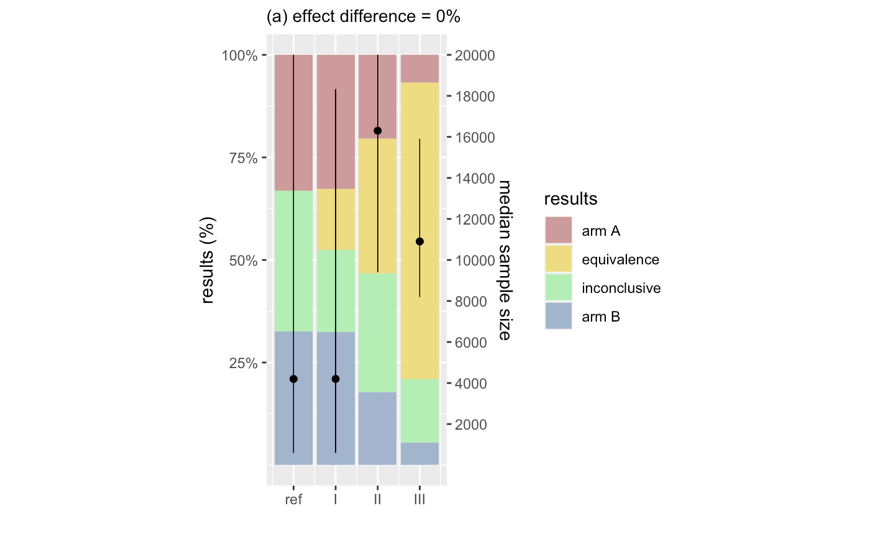

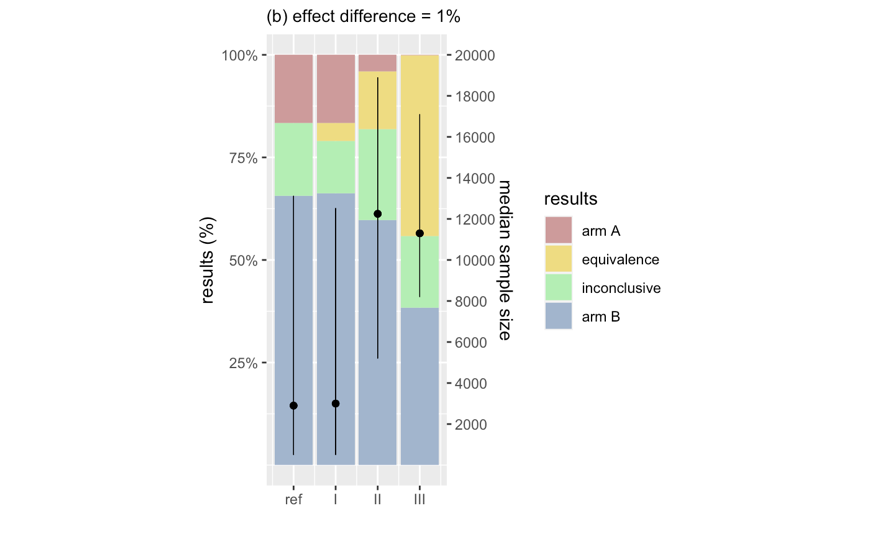

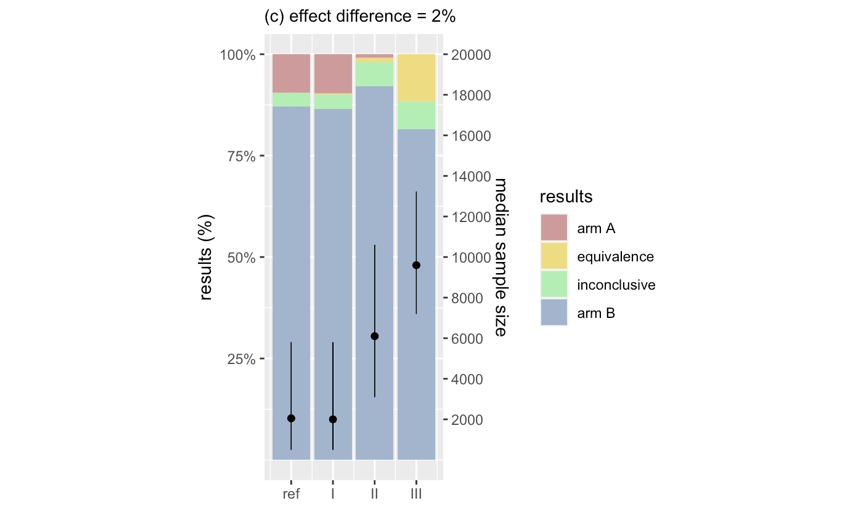


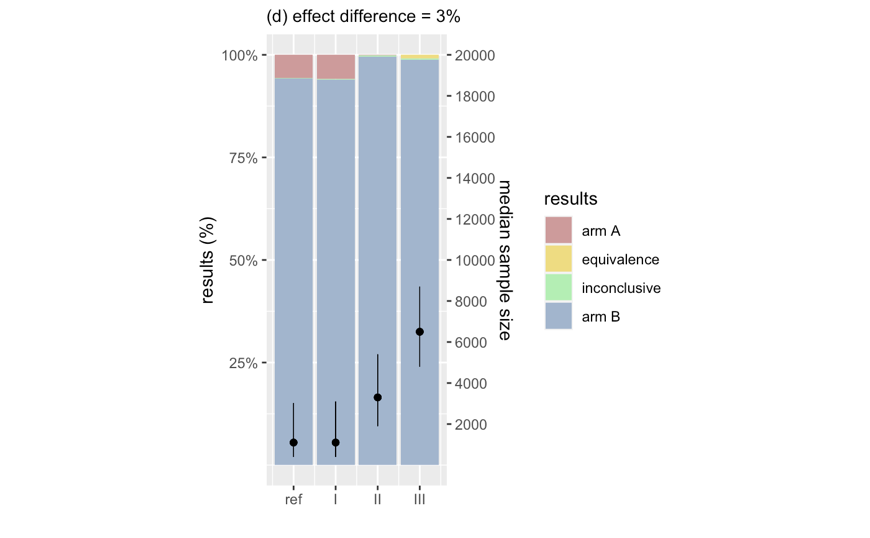

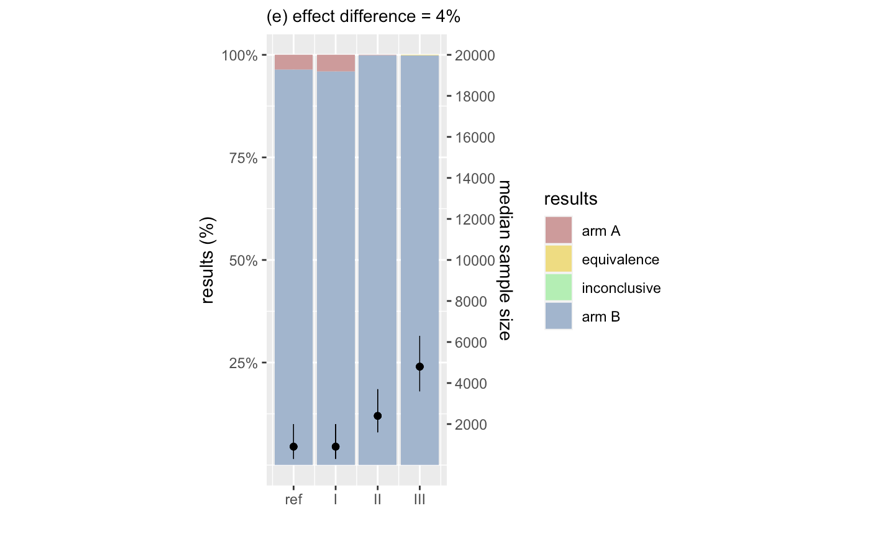

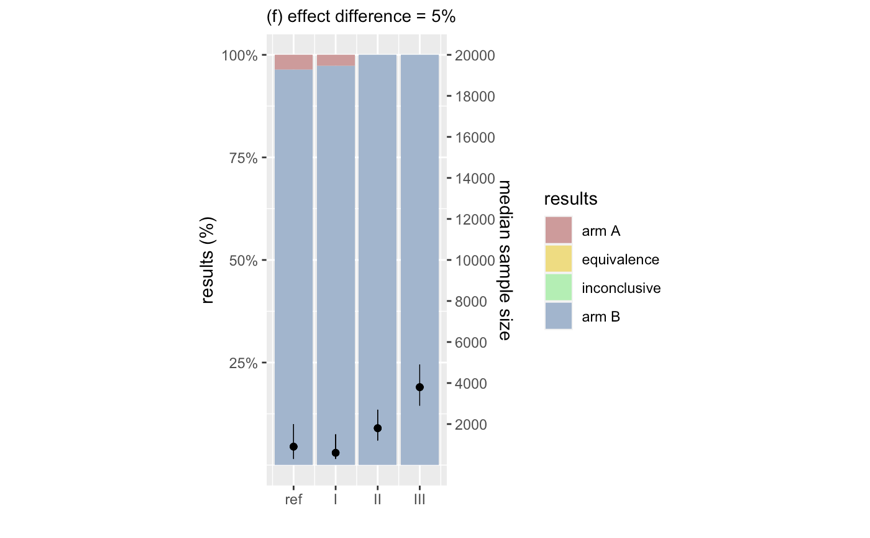


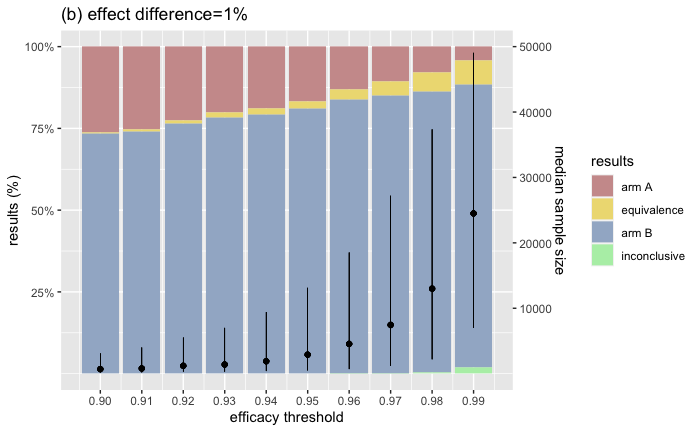


Bars illustrate the trial outcomes, stratified by the magnitude of the true effect difference. Black dots are the median sample sizes of simulated trials, with vertical lines indicating the interquartile ranges. In figure (a), yellow bars represent the proportion of true equivalence outcomes that correctly concluded equivalence between the two arms. In figures (b)-(f), blue bars are the proportions of true efficacy outcomes where the superior arm was correctly identified. Green bars indicate trials that hit the maximum sample size before drawing a conclusion.

| True effect difference | Winning arm identified | | | |  | Sample size | | |
| --- | --- | --- | --- | --- | --- | --- | --- | --- |
| Reference design | | | | | | | | |
|  | **Arm A** | **Arm B** | **Equivalence** | **Inconclusive** |  | **Median (IQR)** | **Minimum** | **Maximum** |
| 0% | 33.2% | 32.6% | **0%** | **34.2%** |  | 4,200 (600; 20,000) | 100 | 20,000 |
| 1% | 16.7% | **65.7%** | 0% | 17.6% |  | 2,900 (500; 13,125) | 100 | 20,000 |
| 2% | 9.5% | **87.2%** | 0% | 3.3% |  | 2,050 (500; 5,800) | 100 | 20,000 |
| 3% | 5.7% | **94.2%** | 0% | 0.1% |  | 1,100 (400; 3,025) | 100 | 20,000 |
| 4% | 3.6% | **96.4%** | 0% | 0% |  | 900 (300; 2,000) | 100 | 14,800 |
| 5% | 2.7% | **97.3%** | 0% | 0% |  | 600 (300; 1,500) | 100 | 9,100 |
| Design (I) | | | | | | | | |
|  | **Arm A** | **Arm B** | **Equivalence** | **Inconclusive** |  | **Median (IQR)** | **Minimum** | **Maximum** |
| 0% | 32.7% | 32.5% | **14.8%** | **20.0%** |  | 4,200 (600; 18,325) | 100 | 20,000 |
| 1% | 16.6% | **66.2%** | 4.5% | 12.7% |  | 3,000 (500; 12,525) | 100 | 20,000 |
| 2% | 9.7% | **86.5%** | 0.2% | 3.6% |  | 2,000 (500; 5,800) | 100 | 20,000 |
| 3% | 5.7% | **94.0%** | 0% | 0.1% |  | 1,100 (400; 3,100) | 100 | 20,000 |
| 4% | 3.6% | **96.0%** | 0% | 0% |  | 900 (300; 2,000) | 100 | 14,600 |
| 5% | 2.7% | **97.3%** | 0% | 0% |  | 600 (300; 1,500) | 100 | 9,100 |
| Design (II) | | | | | | | | |
|  | **Arm A** | **Arm B** | **Equivalence** | **Inconclusive** |  | **Median (IQR)** | **Minimum** | **Maximum** |
| 0% | 20.5% | 17.8% | **32.8%** | **28.9%** |  | 16,300 (9,400; 20,000) | 500 | 20,000 |
| 1% | 4.1% | **59.8%** | 14.0% | 22.1% |  | 12,250 (5,200; 18,900) | 600 | 20,000 |
| 2% | 0.9% | **92.1%** | 1.0% | 6.0% |  | 6,100 (3,100; 10,600) | 500 | 20,000 |
| 3% | 0.2% | **99.6%** | 0% | 0.2% |  | 3,300 (1,900; 5,400) | 300 | 20,000 |
| 4% | 0.1% | **99.9%** | 0% | 0% |  | 2,400 (1,600; 3,700) | 400 | 15,000 |
| 5% | 0% | **100%** | 0% | 0% |  | 1,800 (1,200; 2,700) | 400 | 10,100 |
| Design (III) | | | | | | | | |
|  | **Arm A** | **Arm B** | **Equivalence** | **Inconclusive** |  | **Median (IQR)** | **Minimum** | **Maximum** |
| 0% | 6.8% | 5.5% | **72.2%** | **15.5%** |  | 10,900 (8,200; 15,900) | 2,300 | 20,000 |
| 1% | 0.2% | **38.4%** | 44.0% | 17.4% |  | 11,300 (8,200; 17,100) | 2,300 | 20,000 |
| 2% | 0% | **81.6%** | 11.7% | 6.7% |  | 9,600 (7,200; 13,225) | 2,100 | 20,000 |
| 3% | 0% | **98.8%** | 0.9% | 0.3% |  | 6,500 (4,800; 8,700) | 1,600 | 20,000 |
| 4% | 0% | **99.8%** | 0.2% | 0% |  | 4,800 (3,600; 6,300) | 1,400 | 15,900 |
| 5% | 0% | **100%** | 0% | 0% |  | 3,800 (2,900; 4,900) | 1,200 | 13,000 |

The first column shows the true effect difference of each scenario, and the next four columns show the trial outcomes. The last three columns show the final sample sizes across both arms overall.
