# Supplementary material for: Bayesian adaptive trial designs for evaluating low-risk programmatic changes for quality improvement in health services: a simulation study
Source: BMC Med Res Methodol. 2026 Feb 27;26:75. doi: 10.1186/s12874-026-02780-w (PMC13049811; doi:10.1186/s12874-026-02780-w)
Supplement: Supplementary file 2 — Supplementary Material 2. [file 12874_2026_2780_MOESM2_ESM.docx]

**Title:**

Bayesian adaptive trial designs for data-driven quality improvement in health services: a simulation study

**Journal:**

BMC Medical Research Methodology

**Authors:**

Min Jung Kim^1^, David Prieto-Merino^1,2^, Jennifer Nicholas^1^, Luke Allen^1^, Andrew Bastawrous^3^ , David Macleod^1^

^1^ Faculty of Epidemiology and Population Health, London School of Hygiene & Tropical Medicine, London, UK

^2^ Universidad de Alcalá, Madrid, Spain

^3^ International Centre for Eye Health, Clinical Research Department, London School of Hygiene & Tropical Medicine (LSHTM), London, United Kingdom.

Author information

| **Author** | **Email address** |
| --- | --- |
| Min Jung Kim | min.kim@lshtm.ac.uk |
| David Prieto-Merino | david.prieto@lshtm.ac.uk |
| Jennifer Nicholas | jennifer.nicholas@lshtm.ac.uk |
| Luke Allen | luke.allen@lshtm.ac.uk |
| Andrew Bastawrous | andrew.bastawrous@lshtm.ac.uk |
| David Macleod | david.macleod@lshtm.ac.uk |

Corresponding author

| **Name** | Min Jung Kim |
| --- | --- |
| **Email address** | min.kim@lshtm.ac.uk |
| **Address** | London School of Hygiene and Tropical Medicine  Keppel Street, London, United Kingdom WC1E 7HT |

***Appendix figure 1****. Effects of choice of priors on error rates and sample size*


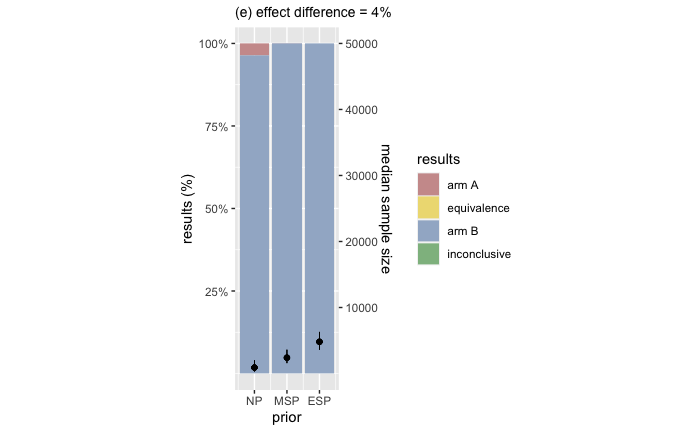

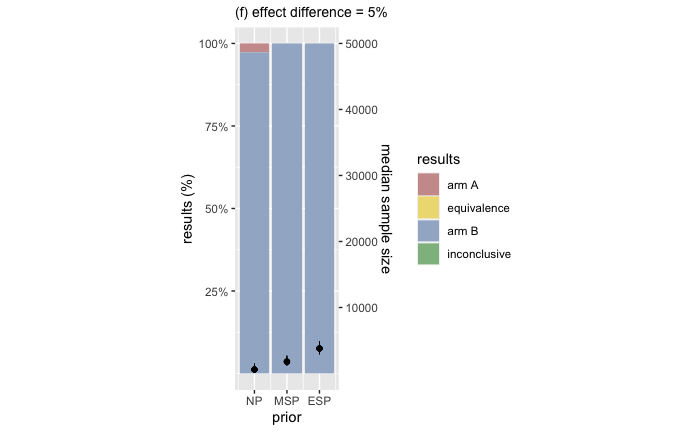


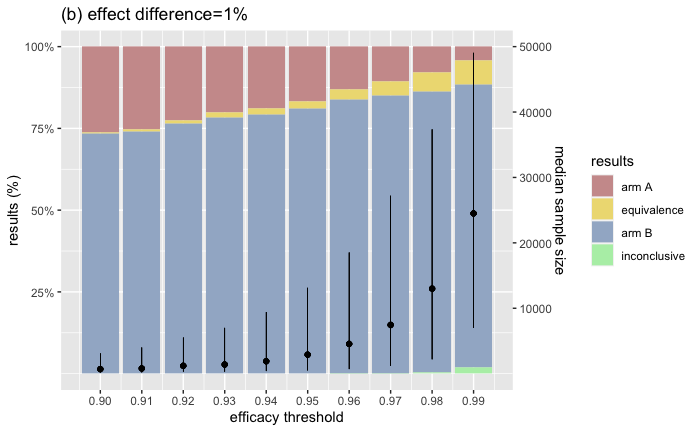


Bars illustrate the trial outcomes, stratified by the magnitude of the true effect difference. Black dots are the median sample sizes of simulated trials, with vertical lines indicating the interquartile ranges. X-axis shows the choice of prior distributions for effect difference, where NP is a neutral prior, MSP is a moderate sceptical prior, and ESP is an extreme sceptical prior. Blue bars are the proportions of true efficacy outcomes where the superior arm was correctly identified.

| True effect difference | Winning arm identified | | |  | Sample size | | |
| --- | --- | --- | --- | --- | --- | --- | --- |
| *Neutral prior* | | | | | | | |
|  | **Arm A** | **Arm B** | **Equivalence** |  | **Median (IQR)** | **Minimum** | **Maximum** |
| 0% | 38.2% | 36.3% | **25.5%** |  | 4,200 (600; 39,300) | 100 | 89,100 |
| 1% | 16.7% | **81.0%** | 2.3% |  | 2,900 (500; 13,125) | 100 | 99,200 |
| 2% | 9.5% | **90.5%** | 0% |  | 2,050 (500; 5,800) | 100 | 47,900 |
| 3% | 5.7% | **94.3%** | 0% |  | 1,100 (400; 3,025) | 100 | 21,400 |
| 4% | 3.6% | **96.4%** | 0% |  | 900 (300; 2,000) | 100 | 14,800 |
| 5% | 2.7% | **97.3%** | 0% |  | 600 (300; 1,500) | 100 | 9,100 |
| *Moderate sceptical prior* | | | | | | | |
|  | **Arm A** | **Arm B** | **Equivalence** |  | **Median (IQR)** | **Minimum** | **Maximum** |
| 0% | 29.5% | 24.8% | **45.7%** |  | 37,300 (8,950; 45,325) | 500 | 90,600 |
| 1% | 4.2% | **90.0%** | 5.8% |  | 12,150 (5,200; 28,925) | 600 | 100,000 |
| 2% | 0.9% | **99.1%** | 0% |  | 6,000 (3,100; 10,600) | 500 | 48,200 |
| 3% | 0.2% | **99.8%** | 0% |  | 3,300 (1,900; 5,500) | 300 | 22,500 |
| 4% | 0.1% | **99.9%** | 0% |  | 2,400 (1,575; 3,600) | 400 | 15,000 |
| 5% | 0% | **100%** | 0% |  | 1,800 (1,200; 2,700) | 400 | 10,100 |
| *Extreme sceptical prior* | | | | | | | |
|  | **Arm A** | **Arm B** | **Equivalence** |  | **Median (IQR)** | **Minimum** | **Maximum** |
| 0% | 17.0% | 12.5% | **70.5%** |  | 34,050 (29,300; 44,100) | 2,300 | 81,600 |
| 1% | 0.2% | **86.1%** | 13.7% |  | 24,350 (13,075; 34,900) | 2,300 | 78,800 |
| 2% | 0% | **99.9%** | 0.1% |  | 10,800 (7,400; 15,700) | 2,100 | 50,100 |
| 3% | 0% | **100%** | 0% |  | 6,500 (4,800; 8,800) | 1,600 | 22,900 |
| 4% | 0% | **100%** | 0% |  | 4,800 (3,600; 6,300) | 1,400 | 15,900 |
| 5% | 0% | **100%** | 0% |  | 3,800 (2,900; 4,900) | 1,200 | 13,000 |

The first column shows the true effect difference of each scenario, and the next three columns show the trial outcomes. The last three columns show the final sample sizes across both arms overall. These designs used different priors. Interim analyses were conducted every 100 participants, and trials were allowed to stop early under two conditions: (a) stop for efficacy if there is at least a 95% posterior probability that the two arms are different by any amount; or (b) stop for equivalence if there is at least a 95% posterior probability that the effect difference between the two arms is less than 1%.

***Appendix figure 2****. Effects of sample size at first interim analysis on error rates and sample size*


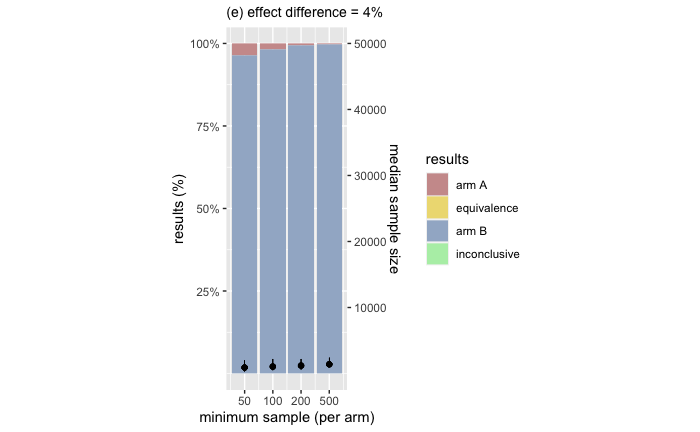

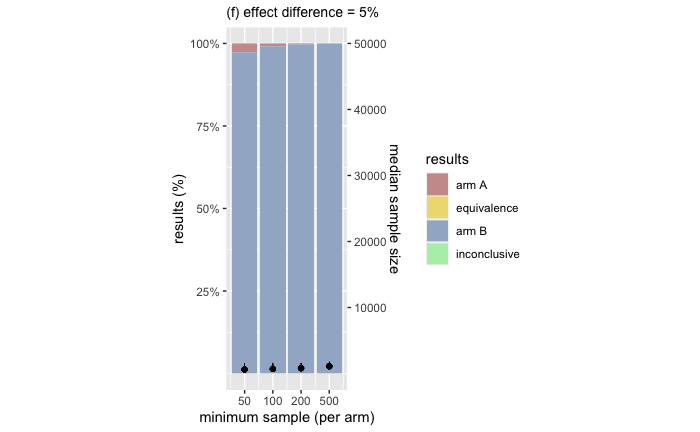


*
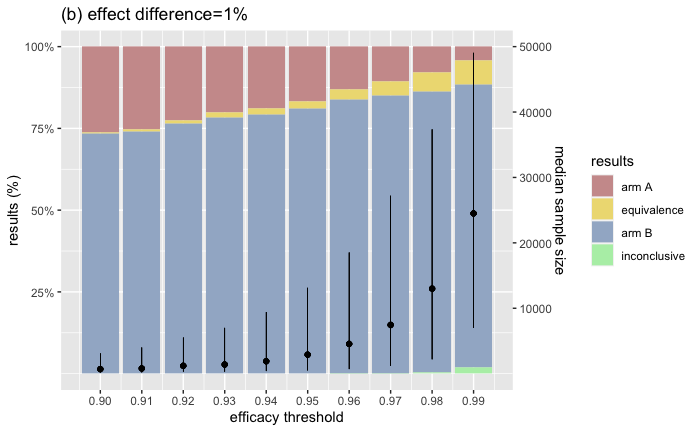
*

Bars illustrate the trial outcomes, stratified by the magnitude of the true effect difference. Black dots are the median sample sizes of simulated trials, with vertical lines indicating the interquartile ranges. X-axis shows the sample size accrued before conducting the first interim analysis. Blue bars are the proportions of true efficacy outcomes where the superior arm was correctly identified.

| True effect difference | Winning arm identified | | |  | Sample size | | |
| --- | --- | --- | --- | --- | --- | --- | --- |
| *N=50 per arm* | | | | | | | |
|  | **Arm A** | **Arm B** | **Equivalence** |  | **Median (IQR)** | **Minimum** | **Maximum** |
| 0% | 38.2% | 36.3% | **25.5%** |  | 4,200 (600; 39,300) | 100 | 89,100 |
| 1% | 16.7% | **81.0%** | 2.3% |  | 2,900 (500; 13,125) | 100 | 99,200 |
| 2% | 9.5% | **90.5%** | 0% |  | 2,050 (500; 5,800) | 100 | 47,900 |
| 3% | 5.7% | **94.3%** | 0% |  | 1,100 (400; 3,025) | 100 | 21,400 |
| 4% | 3.6% | **96.4%** | 0% |  | 900 (300; 2,000) | 100 | 14,800 |
| 5% | 2.7% | **97.3%** | 0% |  | 600 (300; 1,500) | 100 | 9,100 |
| *N=100 per arm* | | | | | | | |
|  | **Arm A** | **Arm B** | **Equivalence** |  | **Median (IQR)** | **Minimum** | **Maximum** |
| 0% | 37.2% | 28.4% | **28.4%** |  | 6,100 (1,000; 40,700) | 300 | 89,100 |
| 1% | 14.2% | **83.4%** | 2.4% |  | 4,100 (900; 14,800) | 300 | 88,100 |
| 2% | 7.2% | **92.8%** | 0% |  | 2,400 (700; 6,525) | 300 | 46,800 |
| 3% | 2.8% | **97.2%** | 0% |  | 1,450 (600; 3,400) | 300 | 21,400 |
| 4% | 1.7% | **98.3%** | 0% |  | 1,050 (500; 2,200) | 300 | 14,800 |
| 5% | 1.0% | **99.0%** | 0% |  | 700 (400; 1,600) | 300 | 9,000 |
| *N=200 per arm* | | | | | | | |
|  | **Arm A** | **Arm B** | **Equivalence** |  | **Median (IQR)** | **Minimum** | **Maximum** |
| 0% | 37.1% | 33.4% | **29.5%** |  | 7,500 (1,400; 41,100) | 500 | 89,300 |
| 1% | 12.8% | **84.7%** | 2.5% |  | 4,800 (1,100; 16,225) | 500 | 96,600 |
| 2% | 5.3% | **94.7%** | 0% |  | 2,750 (900; 6,900) | 500 | 47,900 |
| 3% | 2.5% | **97.5%** | 0% |  | 1,550 (700; 3,500) | 500 | 22,500 |
| 4% | 0.7% | **99.3%** | 0% |  | 1,200 (600; 2,200) | 500 | 14,600 |
| 5% | 0.4% | **99.6%** | 0% |  | 800 (500; 1,600) | 500 | 9,400 |
| *N=500 per arm* | | | | | | | |
|  | **Arm A** | **Arm B** | **Equivalence** |  | **Median (IQR)** | **Minimum** | **Maximum** |
| 0% | 35.5% | 32.0% | **32.5%** |  | 11,300 (2,400; 42,000) | 1,100 | 89,100 |
| 1% | 9.6% | **86.9%** | 3.5% |  | 6,500 (1,900; 19,525) | 1,100 | 96,700 |
| 2% | 2.7% | **97.3%** | 0% |  | 3,500 (1,500; 7,800) | 1,100 | 48,000 |
| 3% | 1.0% | **99.0%** | 0% |  | 1,900 (1,100; 4,000) | 1,100 | 20,900 |
| 4% | 0.3% | **99.7%** | 0% |  | 1,400 (1,100; 2,400) | 1,100 | 14,800 |
| 5% | 0% | **100%** | 0% |  | 1,100 (1,100; 1,800) | 1,100 | 9,600 |

The first column shows the true effect difference of each scenario, and the next three columns show the trial outcomes. The last three columns show the final sample sizes across both arms overall. These designs used different sample size at first interim analysis. A neutral prior was used, and interim analyses were conducted every 100 participants. Trials were allowed to stop early under two conditions: (a) stop for efficacy if there is at least a 95% posterior probability that the two arms are different by any amount; or (b) stop for equivalence if there is at least a 95% posterior probability that the effect difference between the two arms is less than 1%.

***Appendix figure 3****. Effects* *of frequency of analysis on error rates and sample size*


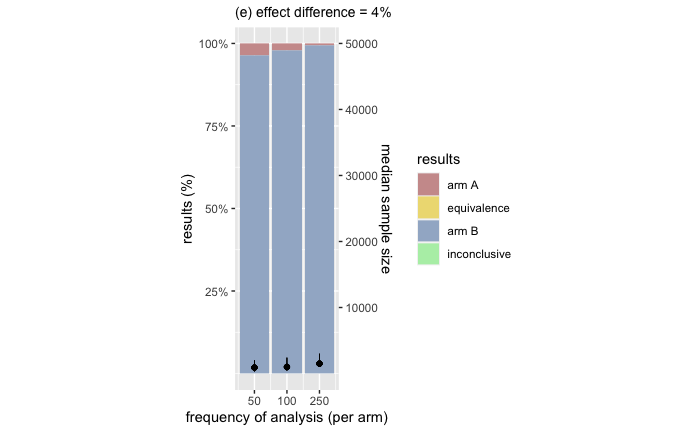

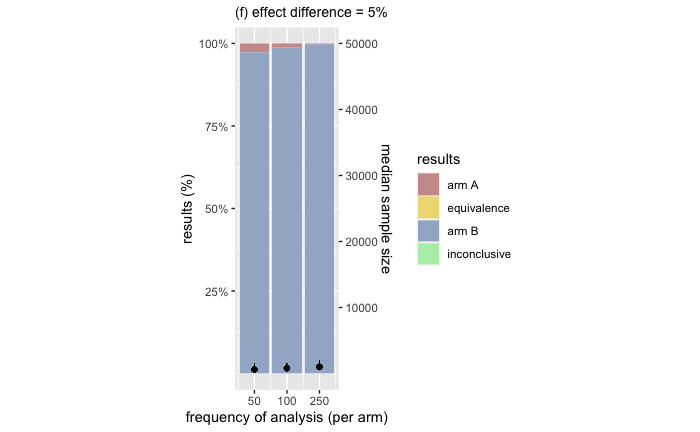


*
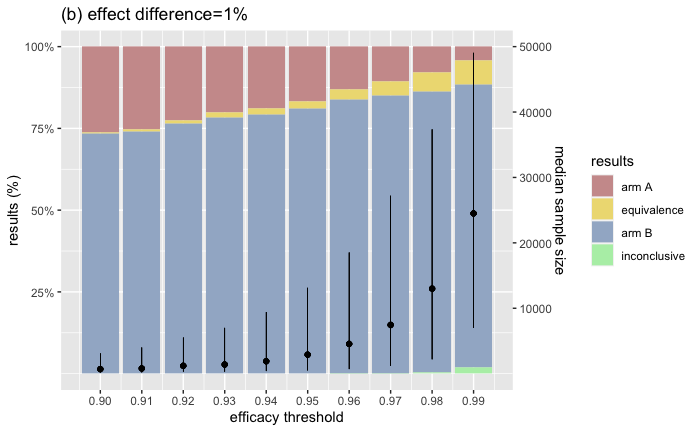
*

Bars illustrate the trial outcomes, stratified by the magnitude of the true effect difference. Black dots are the median sample sizes of simulated trials, with vertical lines indicating the interquartile ranges. X-axis shows the number of extra observations for interim analyses. Blue bars are the proportions of true efficacy outcomes where the superior arm was correctly identified. Green bars indicate trials that hit the maximum sample size before drawing a conclusion.

| True effect difference | | Winning arm identified | | |  |  | Sample size | | |
| --- | --- | --- | --- | --- | --- | --- | --- | --- | --- |
|  | ***N=50 per arm*** | | | | | | | | |
|  | | **Arm A** | **Arm B** | **Equivalence** | **Inconclusive** |  | **Median (IQR)** | **Minimum** | **Maximum** |
| 0% | | 38.2% | 36.3% | **25.5%** | **0%** |  | 4,200 (600; 39,300) | 100 | 89,100 |
| 1% | | 16.7% | **81.0%** | 2.3% | 0% |  | 2,900 (500; 13,125) | 100 | 99,200 |
| 2% | | 9.5% | **90.5%** | 0% | 0% |  | 2,050 (500; 5,800) | 100 | 47,900 |
| 3% | | 5.7% | **94.3%** | 0% | 0% |  | 1,100 (400; 3,025) | 100 | 21,400 |
| 4% | | 3.6% | **96.4%** | 0% | 0% |  | 900 (300; 2,000) | 100 | 14,800 |
| 5% | | 2.7% | **97.3%** | 0% | 0% |  | 600 (300; 1,500) | 100 | 9,100 |
|  | ***N=100 per arm*** | | | | | | | | |
|  | | **Arm A** | **Arm B** | **Equivalence** | **Inconclusive** |  | **Median (IQR)** | **Minimum** | **Maximum** |
| 0% | | 35.1% | 34.2% | **30.7%** | **0%** |  | 9,600 (1,000; 43,800) | 200 | 99,800 |
| 1% | | 10.5% | **84.7%** | 4.8% | 0% |  | 5,400 (800; 20,600) | 200 | 93,800 |
| 2% | | 6.0% | **94.0%** | 0% | 0% |  | 2,400 (800; 6,850) | 200 | 44,200 |
| 3% | | 3.5% | **96.5%** | 0% | 0% |  | 1,600 (600; 3,600) | 200 | 25,600 |
| 4% | | 2.2% | **97.8%** | 0% | 0% |  | 1,000 (400; 2,400) | 200 | 15,600 |
| 5% | | 1.3% | **98.7%** | 0% | 0% |  | 800 (400; 1,600) | 200 | 7,400 |
|  | ***N=250 per arm*** | | | | | | | | |
|  | | **Arm A** | **Arm B** | **Equivalence** | **Inconclusive** |  | **Median (IQR)** | **Minimum** | **Maximum** |
| 0% | | 30.1% | 31.1% | **38.5%** | **0.3%** |  | 25,000 (3,000; 48,000) | 500 | 100,000 |
| 1% | | 8.2% | **87.2%** | 4.6% | 0% |  | 7,000 (2,000; 22,125) | 500 | 86,500 |
| 2% | | 3.2% | **96.8%** | 0% | 0% |  | 3,500 (1,500; 8,000) | 500 | 49,000 |
| 3% | | 2.0% | **98.0%** | 0% | 0% |  | 2,000 (1,000; 4,500) | 500 | 20,500 |
| 4% | | 0.5% | **99.5%** | 0% | 0% |  | 1,500 (1,000; 3,000) | 500 | 11,500 |
| 5% | | 0.3% | **99.7%** | 0% | 0% |  | 1,000 (500; 2,000) | 500 | 8,000 |

The first column shows the true effect difference of each scenario, and the next four columns show the trial outcomes. The last three columns show the final sample sizes across both arms overall. These designs used different frequency of interim analysis. A netural prior was used, and trials were allowed to stop early under two conditions: (a) stop for efficacy if there is at least a 95% posterior probability that the two arms are different by any amount; or (b) stop for equivalence if there is at least a 95% posterior probability that the effect difference between the two arms is less than 1%.

***Appendix figure 4***. *Effects* *of efficacy threshold on error rates and sample size*


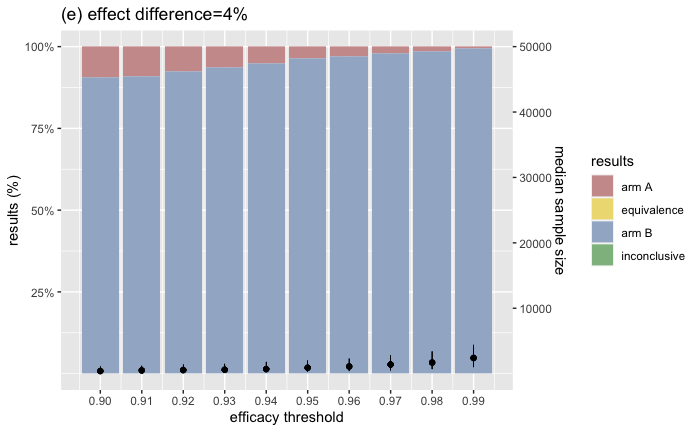

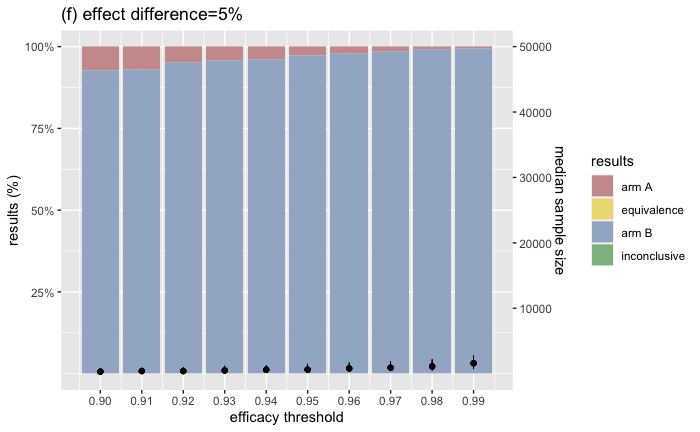


*
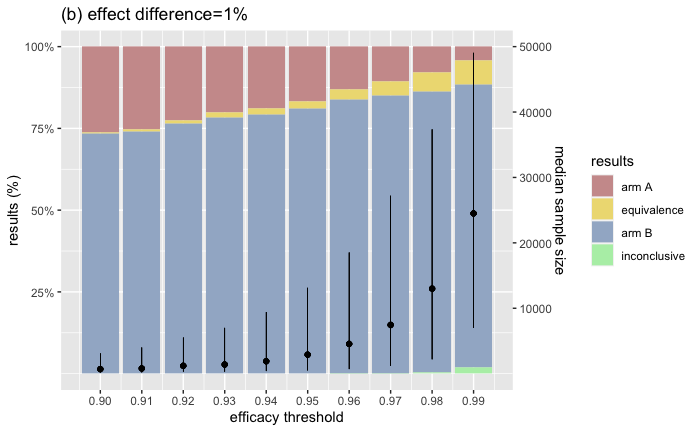
*

Bars illustrate the trial outcomes, stratified by the magnitude of the true effect difference. Black dots are the median sample sizes of simulated trials, with vertical lines indicating the interquartile ranges. X-axis shows the threshold values used to stop trials early for efficacy. Blue bars are the proportions of true efficacy outcomes where the superior arm was correctly identified. Green bars indicate trials that hit the maximum sample size before drawing a conclusion.

| True effect difference | | Winning arm identified | | |  |  | Sample size | | |  |
| --- | --- | --- | --- | --- | --- | --- | --- | --- | --- | --- |
|  | ***S=90%*** | | | | | | | | |  |
|  | | **Arm A** | **Arm B** | **Equivalence** | **Inconclusive** |  | **Median (IQR)** | **Minimum** | **Maximum** |  |
| 0% | | 47.4% | 46.8% | **5.8%** | **0%** |  | 700 (200; 3,300) | 100 | 70,800 |  |
| 1% | | 26.1% | **73.6%** | 0.3% | 0% |  | 700 (200; 3,100) | 100 | 61,700 |  |
| 2% | | 17.6% | **82.4%** | 0% | 0% |  | 600 (200; 2,100) | 100 | 24,400 |  |
| 3% | | 12.0% | **88.0%** | 0% | 0% |  | 500 (200; 1,400) | 100 | 12,300 |  |
| 4% | | 9.4% | **90.6%** | 0% | 0% |  | 400 (200; 1,100) | 100 | 13,000 |  |
| 5% | | 7.2% | **92.8%** | 0% | 0% |  | 300 (100; 800) | 100 | 7,100 |  |
|  | ***S=91%*** | | | | | | | | |  |
|  | | **Arm A** | **Arm B** | **Equivalence** | **Inconclusive** |  | **Median (IQR)** | **Minimum** | **Maximum** |  |
| 0% | | 45.8% | 45.6% | **8.6%** | **0%** |  | 800 (200; 4,700) | 100 | 74,700 |  |
| 1% | | 25.3% | **74.0%** | 0.7% | 0% |  | 800 (200; 4,000) | 100 | 62,100 |  |
| 2% | | 16.3% | **83.7%** | 0% | 0% |  | 700 (200; 2,700) | 100 | 24,400 |  |
| 3% | | 11.2% | **88.8%** | 0% | 0% |  | 600 (200; 1,600) | 100 | 12,400 |  |
| 4% | | 9.1% | **90.9%** | 0% | 0% |  | 500 (200; 1,200) | 100 | 13,000 |  |
| 5% | | 6.8% | **93.2%** | 0% | 0% |  | 400 (100; 900) | 100 | 8,100 |  |
|  | ***S=92%*** | | | | | | | | |  |
|  | | **Arm A** | **Arm B** | **Equivalence** | **Inconclusive** |  | **Median (IQR)** | **Minimum** | **Maximum** |  |
| 0% | | 43.5% | 44.1% | **12.4%** | **0%** |  | 1,200 (300; 7,850) | 100 | 75,000 |  |
| 1% | | 22.4% | **76.5%** | 1.1% | 0% |  | 1,200 (300; 5,525) | 100 | 64,300 |  |
| 2% | | 14.3% | **85.7%** | 0% | 0% |  | 900 (300; 3,100) | 100 | 35,500 |  |
| 3% | | 9.5% | **90.5%** | 0% | 0% |  | 700 (200; 1,900) | 100 | 14,000 |  |
| 4% | | 7.5% | **92.5%** | 0% | 0% |  | 550 (200; 1,400) | 100 | 13,100 |  |
| 5% | | 4.8% | **95.2%** | 0% | 0% |  | 400 (200; 1,000) | 100 | 8,100 |  |
|  | ***S=93%*** | | | | | | | | |  |
|  | | **Arm A** | **Arm B** | **Equivalence** | **Inconclusive** |  | **Median (IQR)** | **Minimum** | **Maximum** |  |
| 0% | | 41.9% | 42.4% | **15.7%** | **0%** |  | 1,650 (300; 14,250) | 100 | 80,700 |  |
| 1% | | 20.2% | **78.4%** | 1.4% | 0% |  | 1,400 (300; 7,000) | 100 | 88,200 |  |
| 2% | | 12.6% | **87.4%** | 0% | 0% |  | 1,200 (300; 4,100) | 100 | 45,500 |  |
| 3% | | 8.4% | **91.6%** | 0% | 0% |  | 800 (200; 2,300) | 100 | 18,600 |  |
| 4% | | 6.4% | **93.6%** | 0% | 0% |  | 600 (200; 1,500) | 100 | 13,100 |  |
| 5% | | 4.3% | **95.7%** | 0% | 0% |  | 500 (200; 1,200) | 100 | 8,300 |  |
|  | ***S=94%*** | | | | | | | | |  |
|  | | **Arm A** | **Arm B** | **Equivalence** | **Inconclusive** |  | **Median (IQR)** | **Minimum** | **Maximum** |  |
| 0% | | 40.1% | 39.8% | **20.1%** | **0%** |  | 2,500 (400; 29,200) | 100 | 89,100 |  |
| 1% | | 18.9% | **79.4%** | 1.7% | 0% |  | 1,900 (400; 9,425) | 100 | 85,100 |  |
| 2% | | 11.7% | **88.3%** | 0% | 0% |  | 1,500 (400; 4,825) | 100 | 46,800 |  |
| 3% | | 7.2% | **92.8%** | 0% | 0% |  | 950 (300; 2,700) | 100 | 20,600 |  |
| 4% | | 5.2% | **94.8%** | 0% | 0% |  | 700 (300; 1,800) | 100 | 13,330 |  |
| 5% | | 3.9% | **96.1%** | 0% | 0% |  | 600 (200; 1,300) | 100 | 9,000 |  |
|  | ***S=95%*** | | | | | | | | |  |
|  | | **Arm A** | **Arm B** | **Equivalence** | **Inconclusive** |  | **Median (IQR)** | **Minimum** | **Maximum** |  |
| 0% | | 38.2% | 36.3% | **25.5%** | **0%** |  | 4,200 (600; 39,300) | 100 | 89,100 |  |
| 1% | | 16.7% | **81.0%** | 2.3% | 0% |  | 2,900 (500; 13,125) | 100 | 99,200 |  |
| 2% | | 9.5% | **90.5%** | 0% | 0% |  | 2,050 (500; 5,800) | 100 | 47,900 |  |
| 3% | | 5.7% | **94.3%** | 0% | 0% |  | 1,100 (400; 3,025) | 100 | 21,400 |  |
| 4% | | 3.6% | **96.4%** | 0% | 0% |  | 900 (300; 2,000) | 100 | 14,800 |  |
| 5% | | 2.7% | **97.3%** | 0% | 0% |  | 600 (300; 1,500) | 100 | 9,100 |  |
|  | ***S=96%*** | | | | | | | | |  |
|  | | **Arm A** | **Arm B** | **Equivalence** | **Inconclusive** |  | **Median (IQR)** | **Minimum** | **Maximum** |  |
| 0% | | 34.5% | 32.4% | **33.1%** | **0%** |  | 9,600 (900; 42,000) | 100 | 91,200 |  |
| 1% | | 13.0% | **83.8%** | 3.1% | 0.1% |  | 4,550 (700; 18,525) | 100 | 100,000 |  |
| 2% | | 7.6% | **92.4%** | 0% | 0% |  | 2,800 (600; 7,200) | 100 | 48,500 |  |
| 3% | | 4.6% | **95.4%** | 0% | 0% |  | 1,400 (500; 3,700) | 100 | 22,500 |  |
| 4% | | 3.1% | **96.9%** | 0% | 0% |  | 1,100 (400; 2,300) | 100 | 15,000 |  |
| 5% | | 2.2% | **97.8%** | 0% | 0% |  | 800 (300; 1,700) | 100 | 10,100 |  |
|  | ***S=97%*** | | | | | | | | |  |
|  | | **Arm A** | **Arm B** | **Equivalence** | **Inconclusive** |  | **Median (IQR)** | **Minimum** | **Maximum** |  |
| 0% | | 30.3% | 26.9% | **42.5%** | **0.3%** |  | 27,250 (1,400; 45,850) | 100 | 100,000 |  |
| 1% | | 10.7% | **84.9%** | 4.3% | 0.1% |  | 7,450 (1,200; 27,200) | 100 | 100,000 |  |
| 2% | | 5.1% | **94.9%** | 0% | 0% |  | 3,900 (1,000; 9,225) | 100 | 52,100 |  |
| 3% | | 3.3% | **96.7%** | 0% | 0% |  | 1,900 (600; 4,400) | 100 | 22,700 |  |
| 4% | | 2.1% | **97.9%** | 0% | 0% |  | 1,400 (500; 2,800) | 100 | 15,400 |  |
| 5% | | 1.4% | **98.6%** | 0% | 0% |  | 900 (400; 1,900) | 100 | 10,100 |  |
|  | ***S=98%*** | | | | | | | | |  |
|  | | **Arm A** | **Arm B** | **Equivalence** | **Inconclusive** |  | **Median (IQR)** | **Minimum** | **Maximum** |  |
| 0% | | 24.7% | 22.0% | **52.4%** | **0.9%** |  | 39,300 (3,700; 50,450) | 100 | 100,000 |  |
| 1% | | 8.0% | **86.0%** | 5.7% | 0.3% |  | 13,000 (2,200; 37,350) | 100 | 100,000 |  |
| 2% | | 3.2% | **96.8%** | 0% | 0% |  | 5,400 (1,500; 11,725) | 100 | 52,900 |  |
| 3% | | 2.3% | **97.7%** | 0% | 0% |  | 2,500 (900; 5,400) | 100 | 23,200 |  |
| 4% | | 1.3% | **98.7%** | 0% | 0% |  | 1,700 (700; 3,400) | 100 | 15,900 |  |
| 5% | | 0.8% | **99.2%** | 0% | 0% |  | 1,100 (500; 2,200) | 100 | 10,300 |  |
|  | | ***S=99%*** | | | | | | | | |
|  | | | **Arm A** | **Arm B** | **Equivalence** | **Inconclusive** |  | **Median (IQR)** | **Minimum** | **Maximum** |
| 0% | | | 15.9% | 14.6% | **66.6%** | **2.9%** |  | 43,350 (24,900; 59,500) | 100 | 100,000 |
| 1% | | | 4.1% | **86.4%** | 7.5% | 2.0% |  | 24,500 (7,000; 49,050) | 100 | 100,000 |
| 2% | | | 1.5% | **98.5%** | 0% | 0% |  | 8,350 (3,100; 16,200) | 100 | 56,000 |
| 3% | | | 1.3% | **98.7%** | 0% | 0% |  | 3,800 (1,500; 6,900) | 100 | 32,700 |
| 4% | | | 0.5% | **99.5%** | 0% | 0% |  | 2,400 (1,000; 4,400) | 100 | 17,500 |
| 5% | | | 0.4% | **99.6%** | 0% | 0% |  | 1,600 (700; 2,800) | 100 | 10,700 |

The first column shows the true effect difference of each scenario, and the next four columns show the trial outcomes. The last three columns show the final sample sizes across both arms overall. These designs used different efficacy thresholds to stop trials for efficacy. Trials were also allowed to stop for equivalence if there is at least a 95% posterior probability that the effect difference between the two arms is less than 1%. A neutral prior was used, and interim analyses were conducted every 100 participants.

***Appendix figure 5***. *Effects* *of equivalence threshold on error rates and sample size*


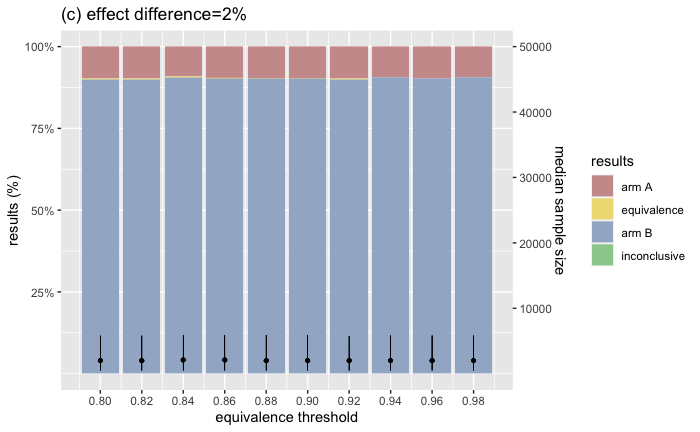

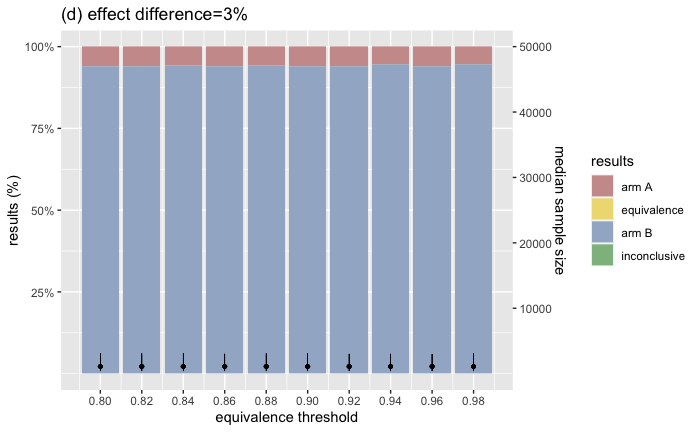

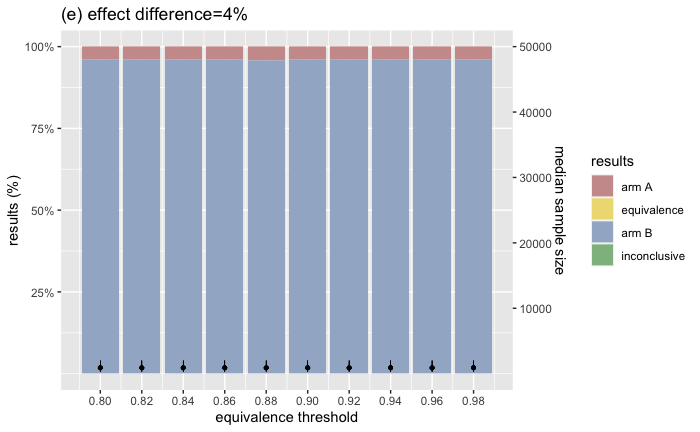

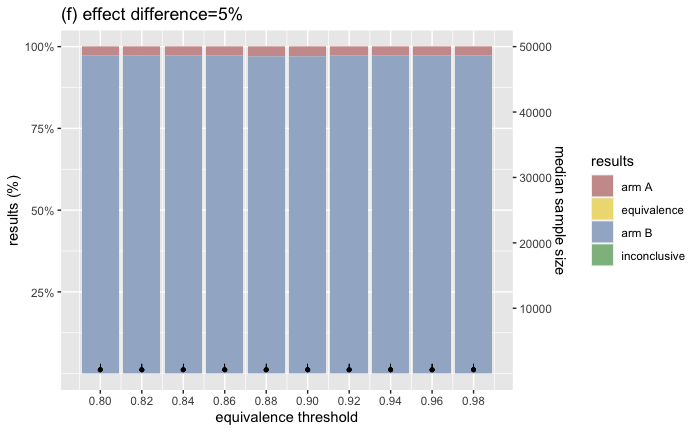


Bars illustrate the trial outcomes, stratified by the magnitude of the true effect difference. Black dots are the median sample sizes of simulated trials, with vertical lines indicating the interquartile ranges. X-axis shows the threshold values used to stop trials early for equivalence. Blue bars are the proportions of true efficacy outcomes where the superior arm was correctly identified. Green bars indicate trials that hit the maximum sample size before drawing a conclusion.

| True effect difference | | Winning arm identified | | |  |  | Sample size | | |  |
| --- | --- | --- | --- | --- | --- | --- | --- | --- | --- | --- |
|  | ***E=80%*** | | | | | | | | |  |
|  | | **Arm A** | **Arm B** | **Equivalence** | **Inconclusive** |  | **Median (IQR)** | **Minimum** | **Maximum** |  |
| 0% | | 35.4% | 34.3% | **30.3%** | **0%** |  | 4,200 (600; 18,325) | 100 | 51,400 |  |
| 1% | | 16.6% | **74.7%** | 8.7% | 0% |  | 3,000 (500; 12,525) | 100 | 50,600 |  |
| 2% | | 9.7% | **90.1%** | 0.2% | 0% |  | 2,000 (500; 5,800) | 100 | 38,500 |  |
| 3% | | 5.9% | **94.1%** | 0% | 0% |  | 1,100 (400; 3,100) | 100 | 22,200 |  |
| 4% | | 4.0% | **96.0%** | 0% | 0% |  | 900 (300; 2,000) | 100 | 14,600 |  |
| 5% | | 2.7% | **97.3%** | 0% | 0% |  | 600 (300; 1,500) | 100 | 9,100 |  |
|  | ***E=82%*** | | | | | | | | |  |
|  | | **Arm A** | **Arm B** | **Equivalence** | **Inconclusive** |  | **Median (IQR)** | **Minimum** | **Maximum** |  |
| 0% | | 35.4% | 34.3% | **30.3%** | **0%** |  | 4,350 (600; 20,100) | 100 | 53,100 |  |
| 1% | | 17.0% | **74.4%** | 8.6% | 0% |  | 3,000 (500; 13,500) | 100 | 61,300 |  |
| 2% | | 9.7% | **90.1%** | 0.2% | 0% |  | 2,000 (500; 5,800) | 100 | 38,500 |  |
| 3% | | 6.0% | **94.0%** | 0% | 0% |  | 1,100 (400; 3,100) | 100 | 21,400 |  |
| 4% | | 3.8% | **96.2%** | 0% | 0% |  | 900 (300; 2,000) | 100 | 14,800 |  |
| 5% | | 2.7% | **97.3%** | 0% | 0% |  | 600 (300; 1,500) | 100 | 9,100 |  |
|  | ***E=84%*** | | | | | | | | |  |
|  | | **Arm A** | **Arm B** | **Equivalence** | **Inconclusive** |  | **Median (IQR)** | **Minimum** | **Maximum** |  |
| 0% | | 35.8% | 34.4% | **29.8%** | **0%** |  | 4,450 (600; 21,700) | 100 | 53,100 |  |
| 1% | | 16.9% | **76.4%** | 7.7% | 0% |  | 3,000 (500; 13,500) | 100 | 61,300 |  |
| 2% | | 9.1% | **90.7%** | 0.2% | 0% |  | 2,100 (500; 5,900) | 100 | 38,500 |  |
| 3% | | 5.8% | **94.2%** | 0% | 0% |  | 1,100 (400; 3,100) | 100 | 21,400 |  |
| 4% | | 3.9% | **96.1%** | 0% | 0% |  | 900 (300; 2,000) | 100 | 14,800 |  |
| 5% | | 2.6% | **97.4%** | 0% | 0% |  | 600 (300; 1,500) | 100 | 9,100 |  |
|  | ***E=86%*** | | | | | | | | |  |
|  | | **Arm A** | **Arm B** | **Equivalence** | **Inconclusive** |  | **Median (IQR)** | **Minimum** | **Maximum** |  |
| 0% | | 35.8% | 34.5% | **29.7%** | **0%** |  | 4,400 (600; 23,900) | 100 | 55,800 |  |
| 1% | | 16.7% | **76.1%** | 7.2% | 0% |  | 3,000 (500; 13,350) | 100 | 62,500 |  |
| 2% | | 9.6% | **90.2%** | 0.2% | 0% |  | 2,100 (500; 5,900) | 100 | 38,500 |  |
| 3% | | 5.9% | **94.1%** | 0% | 0% |  | 1,100 (400; 3,025) | 100 | 22,300 |  |
| 4% | | 3.9% | **96.1%** | 0% | 0% |  | 900 (300; 2,000) | 100 | 14,700 |  |
| 5% | | 2.7% | **97.3%** | 0% | 0% |  | 600 (300; 1,500) | 100 | 9,100 |  |
|  | ***E=88%*** | | | | | | | | |  |
|  | | **Arm A** | **Arm B** | **Equivalence** | **Inconclusive** |  | **Median (IQR)** | **Minimum** | **Maximum** |  |
| 0% | | 36.7% | 34.8% | **28.5%** | **0%** |  | 4,250 (600; 26,300) | 100 | 67,400 |  |
| 1% | | 16.4% | **77.8%** | 5.8% | 0% |  | 3,000 (500; 13,025) | 100 | 70,700 |  |
| 2% | | 9.7% | **90.2%** | 0.1% | 0% |  | 2,000 (500; 5,900) | 100 | 48,500 |  |
| 3% | | 5.7% | **94.3%** | 0% | 0% |  | 1,100 (400; 3,100) | 100 | 22,300 |  |
| 4% | | 4.1% | **95.9%** | 0% | 0% |  | 900 (300; 2,000) | 100 | 14,800 |  |
| 5% | | 2.9% | **97.1%** | 0% | 0% |  | 600 (300; 1,500) | 100 | 9,100 |  |
|  | ***E=90%*** | | | | | | | | |  |
|  | | **Arm A** | **Arm B** | **Equivalence** | **Inconclusive** |  | **Median (IQR)** | **Minimum** | **Maximum** |  |
| 0% | | 37.0% | 35.4% | **27.6%** | **0%** |  | 4,200 (600; 28,400) | 100 | 78,300 |  |
| 1% | | 16.7% | **78.0%** | 5.3% | 0% |  | 3,000 (500; 13,100) | 100 | 70,100 |  |
| 2% | | 9.6% | **90.3%** | 0.1% | 0% |  | 2,000 (500; 5,900) | 100 | 47,300 |  |
| 3% | | 5.9% | **94.1%** | 0% | 0% |  | 1,100 (400; 3,100) | 100 | 22,500 |  |
| 4% | | 3.8% | **96.2%** | 0% | 0% |  | 900 (300; 2,000) | 100 | 14,900 |  |
| 5% | | 2.9% | **97.1%** | 0% | 0% |  | 600 (300; 1,500) | 100 | 9,100 |  |
|  | ***E=92%*** | | | | | | | | |  |
|  | | **Arm A** | **Arm B** | **Equivalence** | **Inconclusive** |  | **Median (IQR)** | **Minimum** | **Maximum** |  |
| 0% | | 37.1% | 35.7% | **27.2%** | **0%** |  | 4,250 (600; 32,025) | 100 | 75,700 |  |
| 1% | | 16.8% | **79.6%** | 3.6% | 0% |  | 2,900 (500; 12,325) | 100 | 70,600 |  |
| 2% | | 9.8% | **90.1%** | 0.1% | 0% |  | 2,000 (500; 5,725) | 100 | 46,900 |  |
| 3% | | 6.0% | **94.0%** | 0% | 0% |  | 1,100 (400; 3,025) | 100 | 21,400 |  |
| 4% | | 4.0% | **96.0%** | 0% | 0% |  | 900 (300; 2,000) | 100 | 14,800 |  |
| 5% | | 2.8% | **97.2%** | 0% | 0% |  | 600 (300; 1,500) | 100 | 9,100 |  |
|  | ***E=94%*** | | | | | | | | |  |
|  | | **Arm A** | **Arm B** | **Equivalence** | **Inconclusive** |  | **Median (IQR)** | **Minimum** | **Maximum** |  |
| 0% | | 37.7% | 36.0% | **26.3%** | **0%** |  | 4,350 (600; 36,500) | 100 | 89,100 |  |
| 1% | | 16.8% | **80.5%** | 2.7% | 0% |  | 3,000 (500; 13,250) | 100 | 84,900 |  |
| 2% | | 9.4% | **90.6%** | 0% | 0% |  | 2,000 (500; 5,825) | 100 | 47,200 |  |
| 3% | | 5.5% | **94.5%** | 0% | 0% |  | 1,100 (400; 3,025) | 100 | 22,500 |  |
| 4% | | 4.0% | **96.0%** | 0% | 0% |  | 900 (300; 2,000) | 100 | 14,800 |  |
| 5% | | 2.8% | **97.2%** | 0% | 0% |  | 600 (300; 1,500) | 100 | 9,100 |  |
|  | ***E=96%*** | | | | | | | | |  |
|  | | **Arm A** | **Arm B** | **Equivalence** | **Inconclusive** |  | **Median (IQR)** | **Minimum** | **Maximum** |  |
| 0% | | 38.3% | 36.0% | **25.7%** | **0%** |  | 4,400 (600; 42,700) | 100 | 90,700 |  |
| 1% | | 16.8% | **81.0%** | 2.1% | 0% |  | 3,000 (500; 13,100) | 100 | 100,000 |  |
| 2% | | 9.6% | **90.4%** | 0% | 0% |  | 2,000 (500; 5,825) | 100 | 46,900 |  |
| 3% | | 6.1% | **93.9%** | 0% | 0% |  | 1,100 (400; 3,000) | 100 | 21,400 |  |
| 4% | | 3.8% | **96.2%** | 0% | 0% |  | 900 (300; 2,000) | 100 | 14,900 |  |
| 5% | | 2.7% | **97.3%** | 0% | 0% |  | 600 (300; 1,500) | 100 | 9,100 |  |
|  | | ***E=98%*** | | | | | | | | |
|  | | | **Arm A** | **Arm B** | **Equivalence** | **Inconclusive** |  | **Median (IQR)** | **Minimum** | **Maximum** |
| 0% | | | 39.0% | 36.5% | **23.9%** | **0.6%** |  | 4,200 (600; 53,300) | 100 | 100,000 |
| 1% | | | 16.5% | **82.5%** | 0.7% | 0.3% |  | 3,100 (500; 13,500) | 100 | 100,000 |
| 2% | | | 9.4% | **90.6%** | 0% | 0% |  | 2,000 (500; 5,825) | 100 | 47,700 |
| 3% | | | 5.5% | **94.5%** | 0% | 0% |  | 1,100 (400; 3,100) | 100 | 20,900 |
| 4% | | | 3.9% | **96.1%** | 0% | 0% |  | 900 (300; 2,000) | 100 | 14,800 |
| 5% | | | 2.7% | **97.3%** | 0% | 0% |  | 600 (300; 1,500) | 100 | 9,100 |

The first column shows the true effect difference of each scenario, and the next four columns show the trial outcomes. The last three columns show the final sample sizes across both arms overall. These designs used different equivalence thresholds to stop trials for equivalence. Trials were also allowed to stop for efficacy if there is at least a 95% posterior probability the two arms are different by any amount. A neutral prior was used, and interim analyses were conducted every 100 participants.

***Appendix figure 6****. Effects of trial design on error rates and sample size*


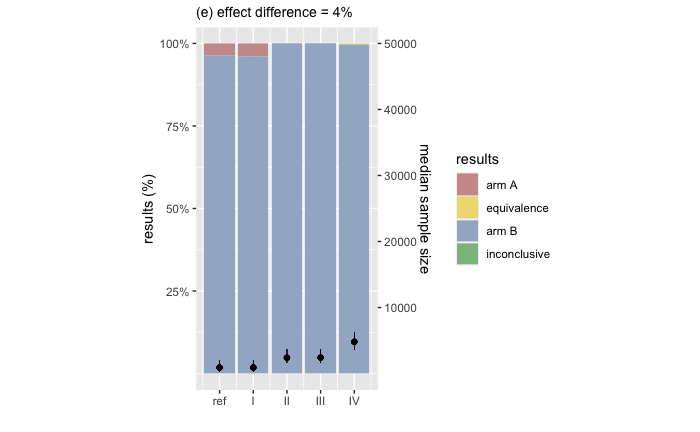

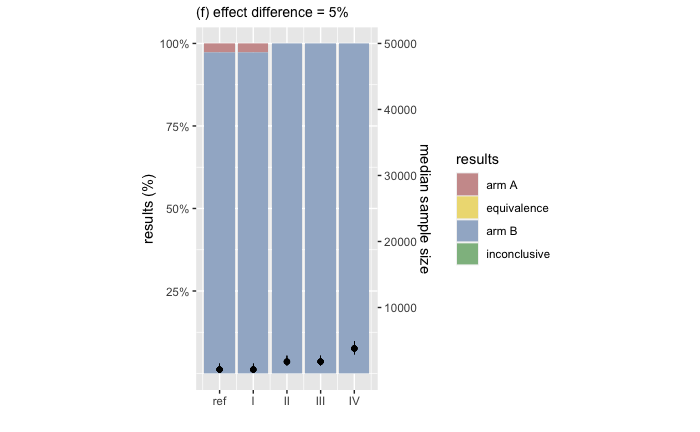


*
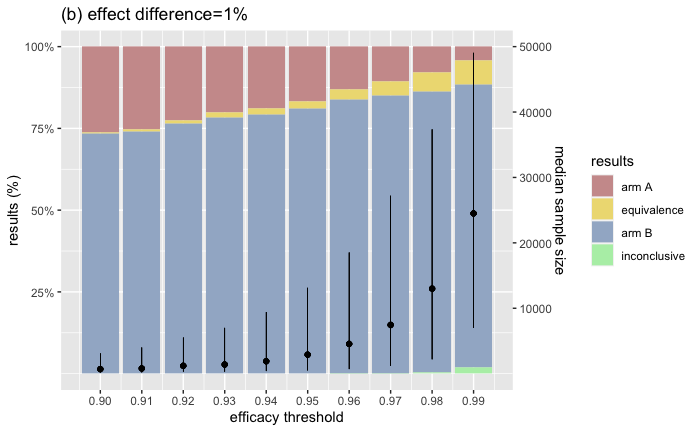
*

Bars illustrate the trial outcomes, stratified by the magnitude of the true effect difference. Black dots are the median sample sizes of simulated trials, with vertical lines indicating the interquartile ranges. In figure (a), yellow bars represent the proportion of trials that correctly concluded equivalence. Blue bars are the proportions of trials that correctly identified the superior arm as the winner.

| True effect difference | Winning arm identified | | |  | Sample size | | |
| --- | --- | --- | --- | --- | --- | --- | --- |
| Reference design | | | | | | | |
|  | **Arm A** | **Arm B** | **Equivalence** |  | **Median (IQR)** | **Minimum** | **Maximum** |
| 0% | 38.2% | 36.3% | **25.5%** |  | 4,200 (600; 39,300) | 100 | 89,100 |
| 1% | 16.7% | **81.0%** | 2.3% |  | 2,900 (500; 13,125) | 100 | 99,200 |
| 2% | 9.5% | **90.5%** | 0% |  | 2,050 (500; 5,800) | 100 | 47,900 |
| 3% | 5.7% | **94.3%** | 0% |  | 1,100 (400; 3,025) | 100 | 21,400 |
| 4% | 3.6% | **96.4%** | 0% |  | 900 (300; 2,000) | 100 | 14,800 |
| 5% | 2.7% | **97.3%** | 0% |  | 600 (300; 1,500) | 100 | 9,100 |
| *design (I)* | | | | | | | |
| True effect difference | **Arm A** | **Arm B** | **Equivalence** |  | **Median (IQR)** | **Minimum** | **Maximum** |
| 0% | 35.4% | 34.3% | **30.3%** |  | 4,200 (600; 18,325) | 100 | 51,400 |
| 1% | 16.6% | **74.7%** | 8.7% |  | 3,000 (500; 12,525) | 100 | 50,600 |
| 2% | 9.7% | **90.1%** | 0.2% |  | 2,000 (500; 5,800) | 100 | 38,500 |
| 3% | 5.9% | **94.1%** | 0% |  | 1,100 (400; 3,100) | 100 | 22,200 |
| 4% | 4.0% | **96.0%** | 0% |  | 900 (300; 2,000) | 100 | 14,600 |
| 5% | 2.7% | **97.3%** | 0% |  | 600 (300; 1,500) | 100 | 9,100 |
| *design (II)* | | | | | | | |
| True effect difference | **Arm A** | **Arm B** | **Equivalence** |  | **Median (IQR)** | **Minimum** | **Maximum** |
| 0% | 24.4% | 20.8% | **54.8%** |  | 16,300 (9,400; 21,200) | 500 | 56,600 |
| 1% | 4.2% | **72.6%** | 23.2% |  | 12,250 (5,200; 18,900) | 600 | 59,900 |
| 2% | 0.9% | **97.8%** | 1.3% |  | 6,100 (3,100; 10,600) | 500 | 38,000 |
| 3% | 0.2% | **99.8%** | 0% |  | 3,300 (1,900; 5,400) | 300 | 22,500 |
| 4% | 0.1% | **99.9%** | 0% |  | 2,400 (1,600; 3,700) | 400 | 15,000 |
| 5% | 0% | **100%** | 0% |  | 1,800 (1,200; 2,700) | 400 | 10,100 |
| *design (III)* | | | | | | | |
| True effect difference | **Arm A** | **Arm B** | **Equivalence** |  | **Median (IQR)** | **Minimum** | **Maximum** |
| 0% | 9.0% | 6.9% | **84.1%** |  | 10,900 (8,200; 15,900) | 2,300 | 45,600 |
| 1% | 0.2% | **50.1%** | 49.7% |  | 11,300 (8,200; 17,100) | 2,300 | 46,400 |
| 2% | 0% | **88.0%** | 12.0% |  | 9,600 (7,200; 13,225) | 2,100 | 34,300 |
| 3% | 0% | **99.1%** | 0.9% |  | 6,500 (4,800; 8,700) | 1,600 | 22,100 |
| 4% | 0% | **99.8%** | 0.2% |  | 4,800 (3,600; 6,300) | 1,400 | 15,900 |
| 5% | 0% | **100%** | 0% |  | 3,800 (2,900; 4,900) | 1,200 | 13,000 |

The first column shows the true effect difference of each scenario, and the next three columns show the trial outcomes. The last three columns show the final sample sizes across both arms overall.

***Appendix figure 7.*** *Mean of posterior distributions of observed effect differences*

| **Mean posterior distributions of observed effect differences** | **Distribution of observed effect differences (i.e. excluding the prior)** |
| --- | --- |
| 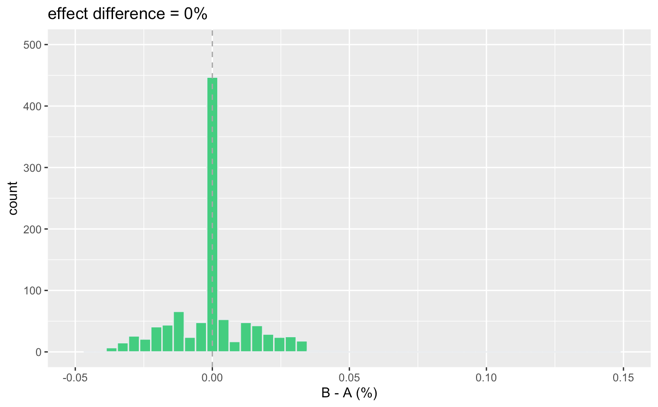 | 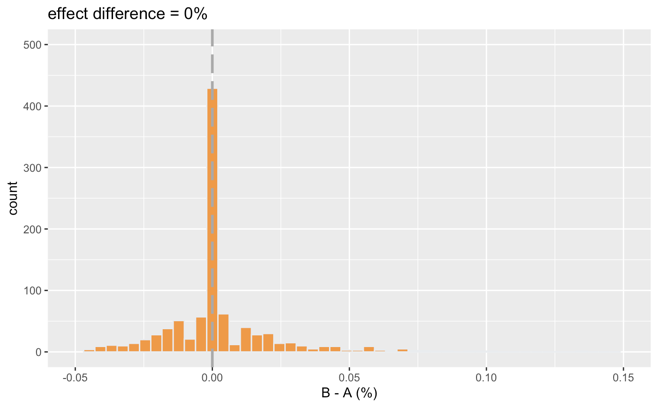 |
| 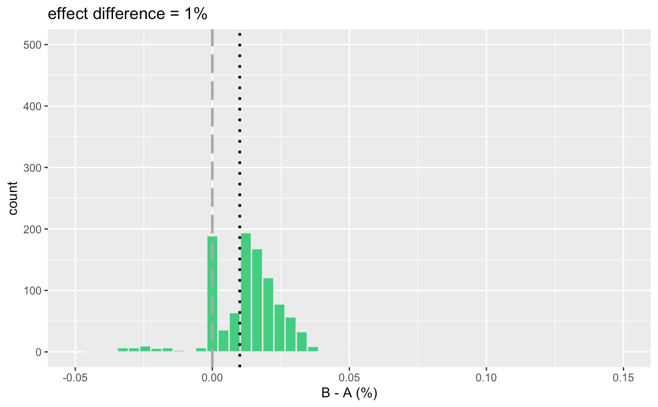 | 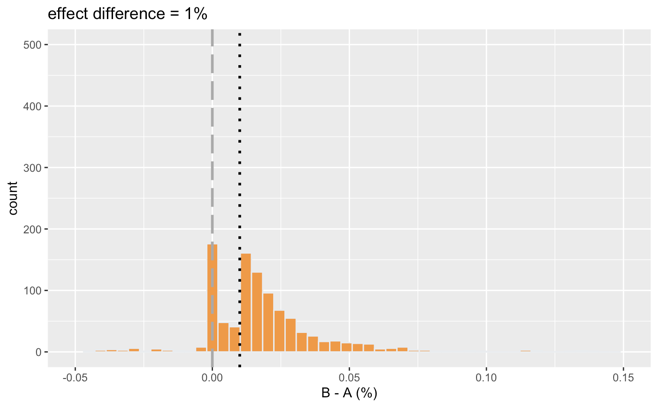 |
| 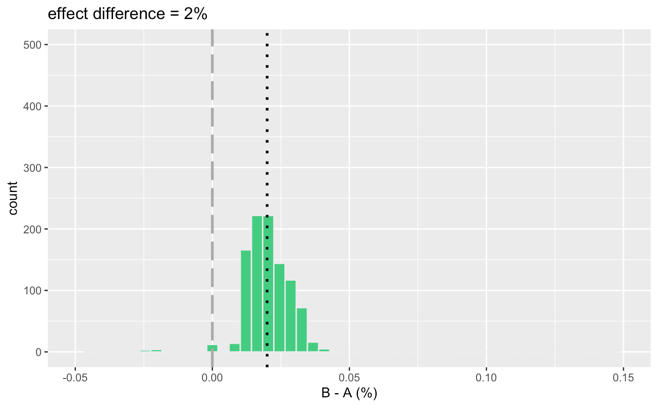 | 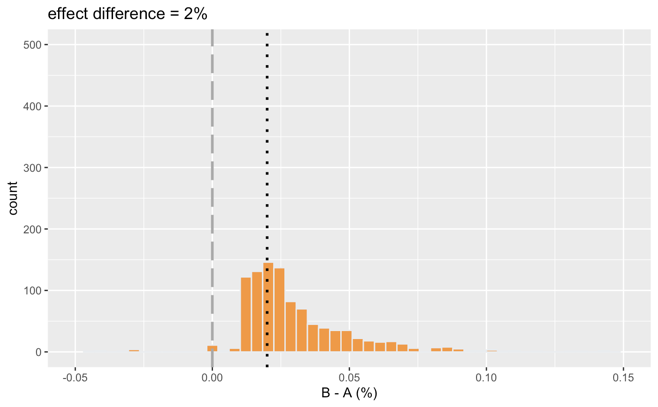 |
| 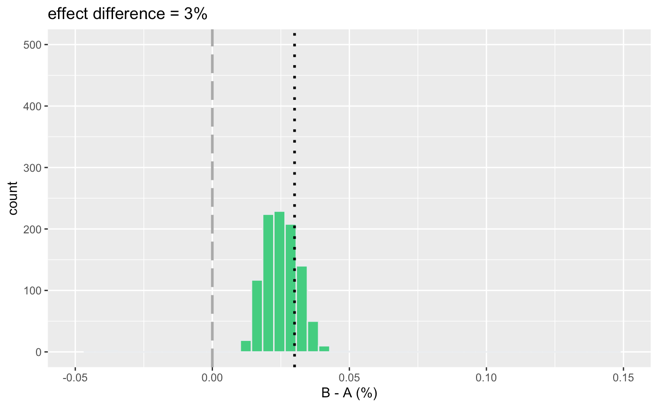 | 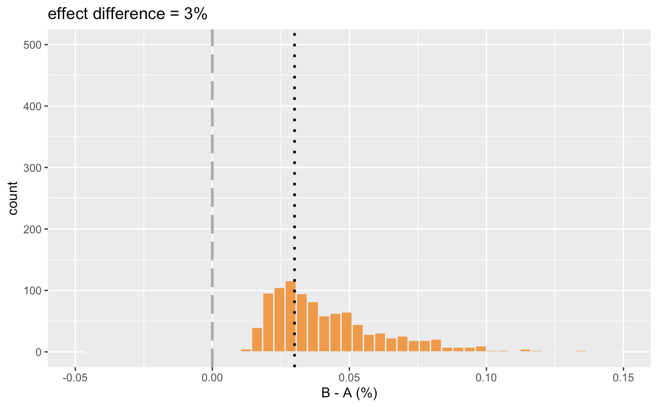 |
| 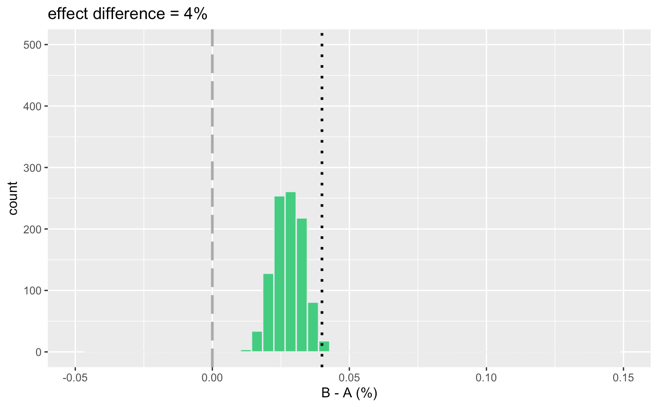 | 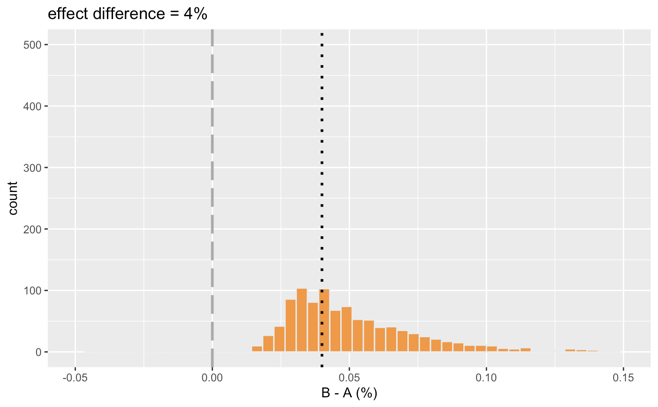 |
| 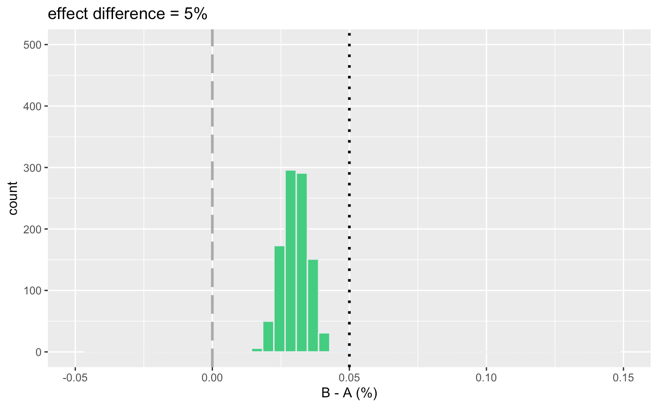 | 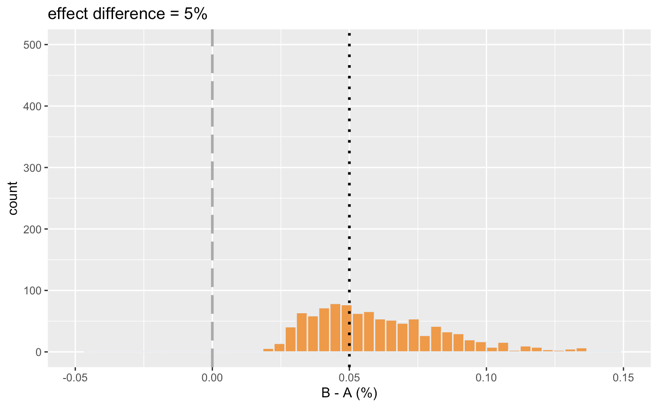 |

The grey dashed line represents no (0%) effect difference, and the black dotted line represents the true effect difference.
